# Identification and characterization of novel marine oxasqualenoid yucatecone against *Naegleria fowleri*

**DOI:** 10.1016/j.ijpddr.2023.05.004

**Published:** 2023-05-29

**Authors:** Iñigo Arberas-Jiménez, Francisco Cen-Pacheco, Javier Chao-Pellicer, Ines Sifaoui, Aitor Rizo-Liendo, Ezequiel Q. Morales, Antonio H. Daranas, Ana R. Díaz-Marrero, José E. Piñero, José J. Fernández, Jacob Lorenzo-Morales

**Affiliations:** a Instituto Universitario de Enfermedades Tropicales y Salud Pública de Canarias (IUETSPC), Universidad de La Laguna (ULL), Avenida Astrofísico Francisco Sánchez s/n, 38206, La Laguna, Tenerife, Spain; b Departamento de Obstetricia y Ginecología, Pediatría, Medicina Preventiva y Salud Pública, Toxicología, Medicina Legal y Forense y Parasitología, Universidad de La Laguna, Tenerife, Spain; c Instituto Universitario de Bio-Orgánica Antonio González (IUBO AG), Universidad de La Laguna (ULL), Avenida Astrofísico Francisco Sánchez 2, 38206 La Laguna, Tenerife, Spain; d Facultad de Bioanálisis, Universidad Veracruzana (UV), Agustín de Iturbide s/n, Centro, Veracruz, 91700, Mexico; e Centro de Investigación Biomédica en Red de Enfermedades Infecciosas (CIBERINFEC), Instituto de Salud Carlos III, Madrid, 28220, Spain; f Instituto de Productos Naturales y Agrobiología (IPNA), Consejo Superior de Investigaciones Científicas (CSIC), Avda. Astrofísico Francisco Sánchez 3, La Laguna, 38206, Tenerife, Spain; g Departamento de Química Orgánica, Universidad de La Laguna (ULL), Avenida Astrofísico Francisco Sánchez s/n, 38203 La Laguna, Tenerife, Spain

**Keywords:** Oxasqualenoids, *Laurencia*, *Naegleria fowleri*, Yucatecone, Primary amoebic meningoencephalitis

## Abstract

*Naegleria fowleri* is an opportunistic protozoan, belonging to the free-living amoeba group, that can be found in warm water bodies. It is causative agent the primary amoebic meningoencephalitis, a fulminant disease with a rapid progression that affects the central nervous system. However, no 100% effective treatments are available and those that are currently used involve the appearance of severe side effects, therefore, there is an urgent need to find novel antiamoebic compounds with low toxicity. In this study, the *in vitro* activity of six oxasqualenoids obtained from the red algae *Laurencia viridis* was evaluated against two different strains of *N*. *fowleri* (ATCC® 30808 and ATCC® 30215) as well as their cytotoxicity against murine macrophages. Yucatecone was the molecule with the highest selectivity index (>2.98 and 5.23 respectively) and it was selected to continue with the cell death type determination assays. Results showed that yucatone induced programmed cell death like responses in treated amoebae causing DNA condensation and cellular membrane damage among others. In this family of oxasqualenoids, it seems that the most significative structural feature to induce activity against *N*. *fowleri* is the presence of a ketone at C-18. This punctual oxidation transforms an inactive compound into a lead compound as the yucatecone and 18-ketodehydrotyrsiferol with IC_50_ values of 16.25 and 12.70 μM, respectively. The assessment of *in silico* ADME/Tox analysis revealed that the active compounds showed good Human Oral Absorption and demonstrate that are found to be within the limit of approved drug parameter range. Hence, the study highlights promising potential of yucatone to be tested for therapeutic use against primary amoebic meningoencephalitis.

## Highlights

• Yucatecone showed the best selectivity index against *N*.*fowleri* of the evaluated oxasqualenoids.
• Yucatecone induced programmed cell death in treated amoebae.
• The presence of a ketone at C-18 seems to be the most significative feature to induce activity in *N*.*fowleri*.
• Yucatecone showed good Human Oral Absorption based on the *in silico* ADME/Tox analysis.

## Graphical abstract

**Figure:**
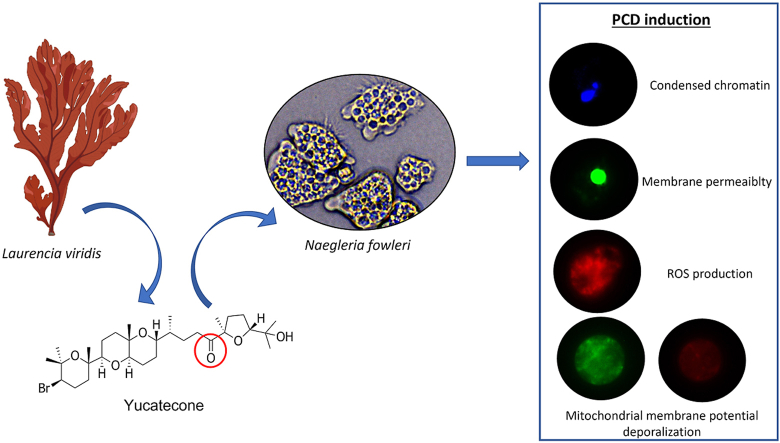

Note: Supplementary data associated with this article.

## 1 Introduction

*Naegleria fowleri* is a free-living amoeba that produces the primary amoebic meningoencephalitis (PAM), a rapidly progressive disease that affects the central nervous system (CNS). Only a 5% of the affected people have survived to the infection. Moreover, this disease is more prevalent in healthy children and young adults (Betanzos et al., 2019; E et al., 2021). *N*. *fowleri* is the only species out of more than 40 of the *Naegleria* genus that is able to infect the human CNS (Zhang and Cheng, 2021). It is a thermophilic amoeba that can tolerate temperatures up to 45 °C (Jahangeer et al., 2020) and can be found in a wide variety of environments, such as freshwater lakes, ponds, domestic water supplies, swimming pools, thermal pools, soil, and dust (Marciano-Cabral and Cabral, 2007).

The infection takes place when the *N*. *fowleri* trophozoites penetrate the nasal cavity, attach to the nasal mucosa, and reach to the to the cribriform plate via the olfactory nerves. Finally, the trophozoites cross the cribriform plate and reach the brain, where they proliferate and cause severe inflammation and an increase in the intracranial pressure (Güémez and García, 2021; Martínez-Castillo et al., 2016; Moseman, 2020). The first symptoms appear within the first 9 days after the water exposure and include bi-frontal headache, seizures or fever that can turn into paralysis, hallucinations, or coma in late stages. The patients' death average is from 1 to 18 days after the first symptom appearance (Rizo-Liendo et al., 2019; Trabelsi et al., 2012).

Most of the infections occur after the performance of recreational water activities like diving or splashing in warm water bodies (Bellini et al., 2020; Pugh and Levy, 2016), although infections after the performance of religious ablution practices or nasal irrigations have also been described (Siddiqui et al., 2016). In addition, infections via cyst-laden dust inhalation have also been suggested. In these, also called “dry-infections”, the cyst transforms into trophozoites once in the nasal passages, before the colonization of the brain. However, they only represent the 6.5% of the total reported PAM cases (Maciver et al., 2020). Despite been detected in all the continents except Antarctica (De Jonckheere, 2011), only about 440 cases have reported worldwide (Abdul Majid et al., 2017; Maciver et al., 2020) since 1965 (first PAM reported case) (Fowler and Carter, 1965). At the present time, 39 countries have reported PAM cases among which the USA, Pakistan and Mexico are the most affected ones (Güémez and García, 2021). However, the rise of the surface water temperature and the alteration of different environmental factors due to the climate change will make increasingly more common to face new PAM cases (Stahl and Olson, 2021).

The diagnosis of PAM is often taken post-mortem due to the rapid progression of the disease (Rizo-Liendo et al., 2020; Schuster and Visvesvara, 2004) and the lack of distinctive clinical features that make it easy to confuse with a viral or bacterial meningoencephalitis (Zhang and Cheng, 2021). In fact, a late diagnosis has been proposed as one of the reasons behind the low surviving rate of the patients (Schuster and Visvesvara, 2004). Thus, the microscopic evaluation of cerebrospinal fluid (where motile *N*.*fowleri* trophozoites can be found) is recommended since a rapid diagnosis is required in order to start the treatment as soon as possible (Mungroo et al., 2019; Siddiqui and Khan, 2014).

The treatments of PAM remains as a major challenge in both developed and developing countries (Jahangeer et al., 2020). Amphotericin B and recently, miltefosine outstand as the most employed drugs in the PAM management (Bellini et al., 2018) and can be used alone or in combination with rifampicin, azithromycin or azoles (Eddie et al., 2022; Heggie and Küpper, 2017). Moreover, intracranial pressure management with therapeutic hypothermia and corticoid administration can be used as adjunctive neuroprotective agents (Cooper et al., 2019; Pugh and Levy, 2016).

The marine environment hosts a wide variety of species that have evolved to survive in severe conditions. These marine organisms have attracted the attention of the scientific community thanks to their capacity to produce bioactive compounds (Karthikeyan et al., 2022). Particularly, the red algae of the *Laurencia* genera have shown to contain numerous active molecules against different protozoa (Desoti et al., 2014; Dos Santos et al., 2010), including some of the free living amoebae (Arberas-Jiménez et al., 2020; García-Davis et al., 2018).

**Fig. 1:**
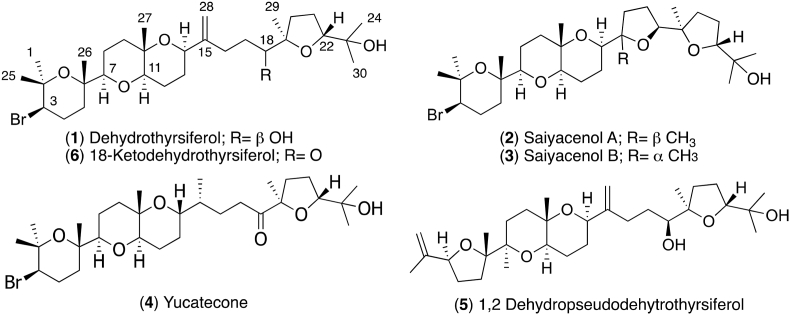
**-** Oxasqualenoid compounds tested against *Naegleria*.

As part of a screening program to identify new marine natural products as scaffolds with anti-*Naegleria* properties, several oxasqualenoid compounds isolated from specimens of the red alga *Laurencia viridis* collected off the coast of the island of Tenerife, Canary Islands, were analyzed (Fig. 1). A study of the activity against two *N*. *fowleri* strains was carried out and the SAR analysis suggested that the importance of the oxidation at carbon C-18 for induction of biological effects.

## 2 Material and methods

### 2.1 Extraction, isolation and identification of oxasqualenoids

Specimens of *Laurencia viridis* were collected along the coast of Paraiso Floral (Tenerife, Canary Islands; 28°07′12’’N, 16°46′45’’W) in springs 2015 and immediately extracted with CHCl_3_:MeOH (1:1) at room temperature. This procedure yielded, after solvent evaporation, 73.5 g of dark-green viscous oil that was first chromatographed on a Sephadex LH-20 column (7 × 50 cm) using CH_3_Cl/MeOH (1:1) as the mobile phase. The fraction 2B (45.4 g) collected between 220 and 365 mL was subsequently processed on a silica gel column (7 × 50 cm) using a linear gradient of *n*-hexane/EtOAc (4:1–1:4). The main metabolite was purified eluting with the 4:1 mixture and all its spectroscopic data were identical to that of dehydrothyrsiferol (**1**, DT) (Gonzalez et al., 1984), while the minor compounds, fraction collected between 366 and 490 mL (9.52 g), were subsequently chromatographed by medium-pressure chromatography employing Lobar LiChroprep-Si 60 with *n*-hexane/acetone (7:3) and then Lobar LiChroprep-RP18 with MeOH/H_2_O (9:1) as eluent. The enriched polyether fraction (4.13 g) was chromatographed on a Lobar LiChroprep Si-60 using DCM/acetone (4:1) and the final purification was done on an HPLC with a μ-Porasil column using the mixtures of DCM/Acetone (17:3) and *n*-hexane/EtOAc (7:3) to afford the pure compounds saiyacenols A (**2**) and B (**3**) (Cen-Pacheco et al., 2012), yucatecone (**4**)(Cen-Pacheco et al., 2021), and 1,2-dehydropseudodehydrothyrsiferol (**5**) (Pacheco et al., 2011).

### 2.2 Chemical transformation of dehydrothyrsiferol (**1**) into 18-ketodehydrothyrsiferol (**6**)

To a solution of dehydrothyrsiferol (**1**) (5 mg, 8.2 μmol) in DCM (0.7 mL) were added 2.5 mg (11.6 μmol) of pyridinium chlorochromate. The resulting mixture was stirred for 3 h at rt. Afterward, the solution was filtered and concentrated to give a solid residue that was chromatographed using HPLC (XTerra column, *n*-hexane:AcOEt 8:2, flow rate 1 mL/min), afforded 18-keto-dehydrothyrsiferol (**6**) (1.4 mg, 2.29 μmol) (yield 28%) (Cen-Pacheco et al., 2021; Lorenzo-Morales et al., 2019).

### 2.3 Amoebic strains and cell maintenance

The activity evaluation of the molecules was carried out in two different *N*. *fowleri* strains (ATCC® 30808™ and ATCC® 30215™) from the American Type Culture Collection (LG Promochem, Barcelona, Spain). The cytotoxicity of the compounds was evaluated using a murine macrophages cell line (ATCC® TIB-67). Cells were grown as previously described (Rizo-Liendo et al., 2020).

### 2.4 *In vitro* activity against *N*. *fowleri* strains

A colorimetric assay based on the alamarBlue® reagent was performed to evaluate the activity of the compounds in two different *N*. *fowleri* strains (ATCC® 30808™ and ATCC® 30215™). The same protocol optimized by Rizo-Liendo et al. was followed in these assays (Rizo-Liendo et al., 2019). Briefly, *N*. *fowleri* trophozoites (2 × 10^5^ cells/mL) were incubated with serial dilutions (in the same culture media) of the compounds in a 96 well microtiter plate. Next, the alamarBlue® reagent was added and the plates were incubated at 37 °C. After 48 h, the fluorescence was read in an EnSpire Multimode Plate Reader (Perkin Elmer, Madrid, Spain) using a wavelength of excitation of 570 nm and a wavelength of emission of 585 nm.

Finally, the inhibitory concentration 50 (IC_50_) and 90 (IC_90_) were calculated with a nonlinear regression analysis with a 95% confidence limit using the SigmaPlot 12.0 sofware (Systat Sofware Inc. London, UK). All experiments were carried out in triplicate and mean values were also calculated.

### 2.5 Cytotoxicity assays

In order to evaluate the cytotoxicity of the compounds a murine macrophages cell line (ATCC® TIB-67) was used. A known concentration of cells (10^5^ cells/mL) was incubated with serial dilutions of the molecules. After that, the alamarBlue® reagent was added and the plates were incubated for 24 h at 37 °C and in a 5% of CO_2_ atmosphere (Arberas-Jiménez et al., 2020). The same alamarBlue® based protocol described in the section above was assessed to obtain de cytotoxic concentration 50 (CC_50_).

### 2.6 *In vitro* activity against *N*. *fowleri* cysts

Cysts of *N*. *fowleri* ATCC® 30808™ strain were used in these assays. Cysts were obtained as previously described and performing the protocol optimized by our group (Arberas-Jiménez et al., 2022). Mature cysts (2 × 10^5^ cells/mL) were incubated with serial dilutions of yucatecone during 24 h at 37 °C. Subsequently, the media was removed and fresh bactocasitone was added with the aim to facilitate the excystation. Finally, the alamarBlue® reagent was added and the plates were incubated for 72 more hours at 37 °C prior to reading the fluorescence and determining the IC_50_ as described in section 2.4.

### 2.7 Cell death type determination

The aim of these assays was to evaluate the presence of different metabolic events that are shown in programmed cell death like process (PCD-like) undergoing cells. In these assays the inhibitory concentration 50 (IC_50_) and the inhibitory concentration 90 (IC_90_) of the yucatecone were used (28.53 ± 5.04 μM and 63.29 ± 0,12 μM respectively) incubating the *N*. *fowleri* ATCC® 30808™ trophozoites (5 × 10^5^ cells/mL) for 24 h. After the treatment of the cells with these concentrations of the molecules, a 50% and a 10%, respectively, of the amoebae in the well remain as viable. Finally, the protocol for each kit was performed following manufacturers’ instructions. Moreover, the percentage of stained cells after the incubation of the treated and non-treated cells with each kit and the ratio of fluorescence between the aggregate and monomer forms of the JC-1 was evaluated. For this, the EVOS M5000 Cell Imaging System (Life Technologies, Madrid, Spain) was used and each experiment was performed in triplicate. In each experiment five different images were evaluated with a minimum number of cells of 80.

#### 2.7.1 Double stain assay for chromatin condensation detection

A double stain detection kit Hoechst 33342/propidium iodide (PI) (Life Technologies, Madrid, Spain) and an EVOS M5000 Cell Imaging System (Life Technologies, Madrid, Spain) were used in this experiment. The assay was performed following manufacturer's instruction.

This kit enables to distinguish between three different group of cells: A light blue fluorescence corresponds to live cells whereas in the treated cells an intense blue fluorescence is shown. Finally, red fluorescence appears in dead cells as the IP binds to their DNA.

#### 2.7.2 Plasmatic membrane permeability

The SYTOX Green kit (Life Technologies, Madrid, Spain) was used for the evaluation of the cellular plasmatic membrane damage. This stain binds to the DNA of those cells who have suffered alterations in the permeability of the plasmatic membrane and emits a high green fluorescence. Hence, no green fluorescence can be shown in healthy cells. This assay was performed following manufacturer's instruction and modified by Sifaoui et al. (2018). An EVOS M5000 Cell Imaging System (Life Technologies, Madrid, Spain) was used to obtain the images.

#### 2.7.3 Generation of intracellular reactive oxygen species (ROS)

The CellROX Deep Red fluorescent assay (Invitrogen, Termo Fisher Scientifc, Madrid, Spain) was used to perform the ROS levels evaluation. After the cell incubation with the yucatecone the stain was added and incubated for half an hour in the dark. Finally, images were obtained with an EVOS M5000 Cell Imaging System (Life Technologies, Madrid, Spain). The presence of ROS is detected by intense red fluorescence.

#### 2.7.4 Mitochondrial membrane potential evaluation

The mitochondrial membrane potential decrease was determined using the JC-1 Mitochondrial Membrane Potential Assay Kit (Cayman Chemicals, Vitro SA, Madrid, Spain). The JC-1 dye presents potential-dependent accumulation in mitochondria. At high membrane potentials (healthy cells) JC-1 dye accumulates as “J-aggregates” in the mitochondria and emits red fluorescence whereas at low membrane potentials (cells undergoing PCD-like responses) the dye is presented in monomeric form in the cytoplasm, emitting green fluorescence. Hence, mitochondrial membrane depolarization is indicated by a decrease in the red/green fluorescence intensity ratio.

The assay was performed incubating the cells with the IC_90_ of yucatecone at 37 °C during 24 h. Treated cells were then incubated with the JC-1 dye for 30 min. Finally, the results were observed in the EVOS M5000 Cell Imaging System (Life Technologies, Madrid, Spain).

#### 2.7.5 ATP detection

The ATP level measurement was performed with the Celltiter-Glo® Luminescent Cell Viability Assay (Promega Biotech Ibérica, Madrid, Spain). The assay was performed after the incubation of the *N*. *fowleri* trophozoites with the IC_90_ of yucatecone and following manufacturer's indications.

### 2.8 Molecular modelling analysis

A conformational study is carried out over the compounds **1**, **4** and **6** using a mixed torsional/low-mode sampling search method as implemented in Schrödinger Suites v. 2022-2 (Schrödinger, 2021). The search is done over 5000 iterations using OPLS4, water as solvent and TNGC method. Conformers with energy over 21 kJ/mol (5.2 kcal/mol) are discarded. From that search 832 conformers of compound **1**, 2506 conformers of compound **4** and 2061 conformers of compound **6** were obtained and classified using X-Cluster (Schrödinger, 2021) software as implemented in Schrödinger and reduced to the main conformers of each compounds: 4 conformers to **1**, 6 conformers to **6** and 10 conformers to **4**. More detailed information about conformers analysis is included at the Supporting Information.

For the reduced set of conformers obtained for each compound we run DFT (Parr and Yang, 1995b; Ziegler, 1991) calculations using the B3LYP-D3 (Becke, 1988; Kohn et al., 1996) functional and 6-31G++** basis set, calculated with Jaguar module of Schrödinger Suites v. 2022-2 (Chattaraj et al., 2006; Schrödinger, 2021). All minima were fully characterized by harmonic frequency analysis (McIver and Komornicki, 1972). The solvent effect in DFT calculations was evaluated by means of the Polarizable Continuum model (PCM) using water as solvent. From those DFT calculations, obtained molecular properties are shown in Table 2.

### 2.9 In silico ADME/Tox analysis

The compounds **1**, **4** and **6** were submitted to *in silico* pharmacokinetic properties prediction by using the graphical interface Maestro and QikProp module of Schrödinger Suites 2022-2 (Schrödinger, 2021). The results are included at the Supporting Information.

## 3 Results and discussion

### 3.1 *In vitro* activity of *Laurencia viridis* derivatives against *N*. *fowleri*

The *in vitro* activity results are summarized in Table 1. A set of natural oxaesqualenoids were tested in this study, among which yucatecone was the most active natural product against *N*. *fowleri*, showing inhibitory concentration 50 (IC_50_) values of 28.53 ± 5.04 μM (Figure S14). Interestingly, this molecule proved to be more active against the clinical strain (ATCC® 30215™) with 16.25 ± 1.23 μM (Figure S15). Regarding the cytotoxicity against murine macrophages (J774A.1), yucatecone showed a cytotoxic concentration 50 (CC_50_) above 85 μM (Table 1).

**Table 1:** Inhibitory concentration 50 (IC_50_) of *Laurencia viridis* isolated compounds against the trophozoite stage of *N*. *fowleri* ATCC® 30808™ and ATCC® 30215™ strains. The cytotoxic concentration 50 (CC_50_) against a murine macrophages cell line (J774A.1) is also shown. Results are expressed as mean concentration ± standard deviation and were conducted in triplicate. The selectivity index (SI) of the active compounds against both strains are indicated. N/A indicates that no activity was observed. The results of two reference drugs (amphotericin B and Miltefosine) for the PAM treatment are also shown.

Compound	*N. fowleri* ATCC® 30808™ IC_50_ (μM)	*N. fowleri* ATCC® 30215™ IC_50_ (μM)	Murine macrophages CC_50_ (μM)	*N. fowleri* ATCC® 30808™ SI (CC_50_/IC_50_)	*N.fowleri* ATCC® 30215™ SI (CC_50_/IC_50_)
DT (1)	N/A	–	–	–	–
Saiyacenol A (2)	N/A	–	–	–	–
Saiyacenol B (3)	N/A	–	–	–	–
Yucatecone (4)	28.53 ± 5.04	16.25 ± 1.23	>85	>2.98	>5.23
1,2-PseudoDT (5)	N/A	–	–	–	–
18-KetoDT (6)	15.33 ± 2.82	12.70 ± 2.64	40.03 ± 1.06	2.61	3.17
Amphotericin B	0.12 ± 0.03	0.16 ± 0.02	>200	>1652.89	>1204.82
Miltefosine	38.74 ± 4.23	81.57 ± 7.23	127,89 ± 8.85	3.30	1.57

**Table 2:** Calculated values of Energy in aqueous solution (PCM model), Solvation Energery; Gap: E_HOMO_ – E_LUMO_; η: Global hardness; μ: Chemical Potential; ω: Global Electrophilicity Index and ΔNmax: Maximum Number of Accepted Electrons for all conformers, Dipole Moment and Polarizability. The structures show the most stable conformer (bold).

Conformer	E. Solution (a.u.)	E. Solvation (kcal/mol)	E_HOMO_ (a.u.)	E_LUMO_ (a.u.)	Gap (-eV)	η (a.u.)	μ (a.u.)	ω (a.u.)	ΔN_max_ (a.u.)	Dipole M (D)	Polarizability	%
												
1.1	−4120.731621	−14.25	−0.248120	−0.022610	6.14	0.225510	−0.135365	0.040627	0.600262	5.3323	493.5364	28.98
1.2	−4120.731541	−13.90	−0.246990	−0.022440	6.11	0.224550	−0.134715	0.040410	0.599933	6.7347	492.0914	28.73
1.3	−4120.729121	−14.53	−0.248000	−0.022400	6.14	0.225600	−0.135200	0.040512	0.599291	3.5320	492.9183	22.24
1.4	−4120.728144	−14.94	−0.247320	−0.022740	6.11	0.224580	−0.135030	0.040594	0.601256	3.7950	492.8380	20.05
4.1	−4120.760528	−15.29	−0.246320	−0.034090	5.77	0.212230	−0.140205	0.046312	0.660628	5.7612	489.3964	12.40
4.2	−4120.758983	−16.15	−0.246410	−0.032600	5.82	0.213810	−0.139505	0.045512	0.652472	4.6369	488.1540	10.53
4.3	−4120.758785	−17.55	−0.245300	−0.032500	5.79	0.212800	−0.138900	0.045332	0.652726	10.2487	488.6704	10.31
4.4	−4120.758720	−17.32	−0.246530	−0.035440	5.74	0.211090	−0.140985	0.047081	0.667890	6.8362	488.0259	10.24
4.5	−4120.758707	−15.42	−0.245240	−0.036010	5.69	0.209230	−0.140625	0.047258	0.672107	7.2079	489.2878	10.22
4.6	−4120.758662	−17.04	−0.246320	−0.029610	5.90	0.216710	−0.137965	0.043917	0.636634	9.4034	488.9467	10.17
4.7	−4120.758199	−16.21	−0.245770	−0.032360	5.81	0.213410	−0.139065	0.045310	0.651633	8.3206	488.1438	9.69
4.8	−4120.757763	−16.10	−0.245040	−0.028350	5.90	0.216690	−0.136695	0.043116	0.630832	7.9528	488.9707	9.25
4.9	−4120.757511	−16.86	−0.246500	−0.030300	5.88	0.216200	−0.138400	0.044298	0.640148	6.8617	488.3563	9.01
4.10	−4120.756612	−15.25	−0.244010	−0.028840	5.85	0.215170	−0.136425	0.043249	0.634034	8.2932	489.6233	8.19
6.1	−4119.517770	−16.60	−0.248300	−0.037640	5.73	0.210660	−0.142970	0.048515	0.678677	3.7742	487.8067	18.72
6.2	−4119.517161	−17.31	−0.248130	−0.037010	5.74	0.211120	−0.142570	0.048139	0.675303	7.4559	489.4127	17.55
6.3	−4119.517019	−17.26	−0.248080	−0.031720	5.89	0.216360	−0.139900	0.045230	0.646608	9.2619	488.3232	17.29
6.4	−4119.516465	−16.69	−0.247880	−0.033690	5.83	0.214190	−0.140785	0.046268	0.657290	7.3222	487.8732	16.30
6.5	−4119.515795	−17.80	−0.247290	−0.031600	5.87	0.215690	−0.139445	0.045076	0.646507	7.0296	488.2173	15.19
6.6	−4119.515650	−16.85	−0.247790	−0.033980	5.82	0.213810	−0.140885	0.046416	0.658926	9.6827	487.9737	14.95

In view of this results, yucatecone stands out as the best molecule in the study. In fact, the calculated selectivity index (Table 1) of this compound against the ATCC® 30215™ strain is over 3-fold the value obtained for the reference drug miltefosine whereas the values against the ATCC® 30808™ are similar. Therefore, yucatecone was selected to carry on further studies.

### 3.2 *In vitro* activity of yucatecone against *N*. *fowleri* cysts

The *in vitro* activity assays of yucatecone against the resistant phase of *N*. *fowleri* showed an IC_50_ value of 9.43 ± 1.48 μM (Figure S16), been even more active than against the trophozoite stage of the amoeba.

### 3.3 Yucatecone induces chromatin condensation in *N*. *fowleri*

**Fig. 2:**
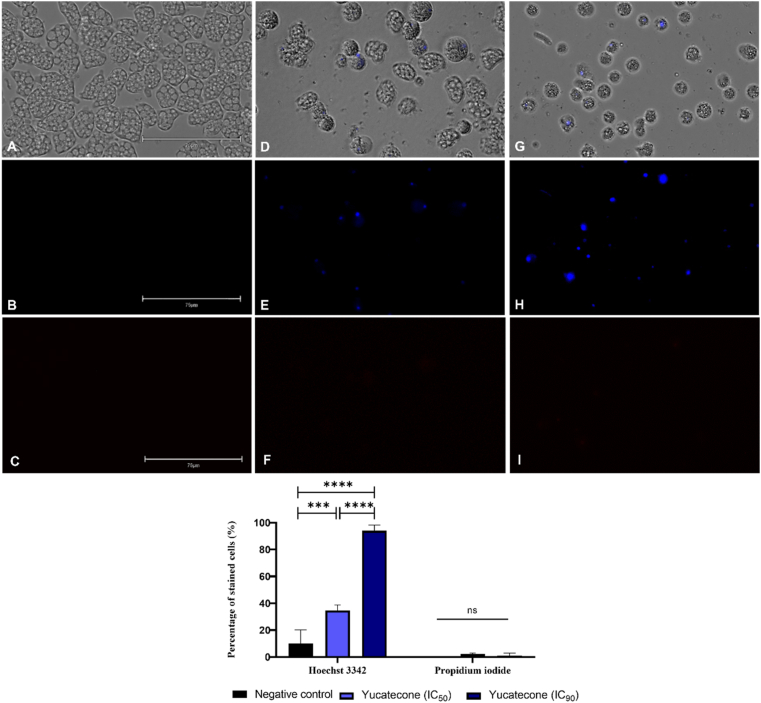
*N. fowleri* cells incubated with the IC_50_ (**D**–**F**) and the IC_90_ of yucatecone after 24 h (**G**–**I**), negative control (**A**–**C**). Non-treated trophozoites show no fluorescence (**B**) while treated amoebae show an intense blue fluorescence (**E** and **H**). Overlay channel (**A**, **D** and **G**); Hoechst channel (**B**, **E** and **H**), and propidium iodide channel (**C**, **F** and **I**). Images (x40) are representative of the cell population observed in the EVOS M5000 Cell Imaging System, Life Technologies, Spain. (Scale bar: 75 μm). The bar graph represents the mean value and the SD of the percentage of stained cells after the incubation of the treated and non-treated cells with the Hoechst 33342 and PI stains. The experiment was carried out in triplicate and every time five different images (x40) were analyzed in the EVOS™ M5000 Software (Invitrogen by Thermo Fisher Scientific). Differences between the values were assessed using one-way analysis of variance (ANOVA) ***p < 0.001; ****p < 0.0001 significance, ns: non significance. (For interpretation of the references to color in this figure legend, the reader is referred to the Web version of this article.)

The performed double stain assay with Hoechst 33342 and PI reactives showed that yucatecone induces chromatin condensation in *N*. *fowleri* trophozoites after the incubation of the cells with the IC_50_ (killing half of the amoebae in the well) and the IC_90_ (10% of the trophozoite remain as viable) of the compound. As it can be seen in Fig. 2 and S17 a bright blue nucleus is shown in treated cells while no fluorescence is emitted in non-treated control cells. Additionally, after the PI assay no fluorescence is shown in the cells after the treatment with yucatecone suggesting an early apoptotic stage. After performing the one-way analysis of variance, it was determined that differences between the percentage of stained cells with the Hoechst 33342, of the negative control and cells treated with both concentrations of yucatecone were statistically significant (***p < 0.001 and ****p < 0.0001, respectively). However, no statistical difference could be observed between the different group of cells after incubating them with the PI.

### 3.4 Plasma membrane permeability

**Fig. 3:**
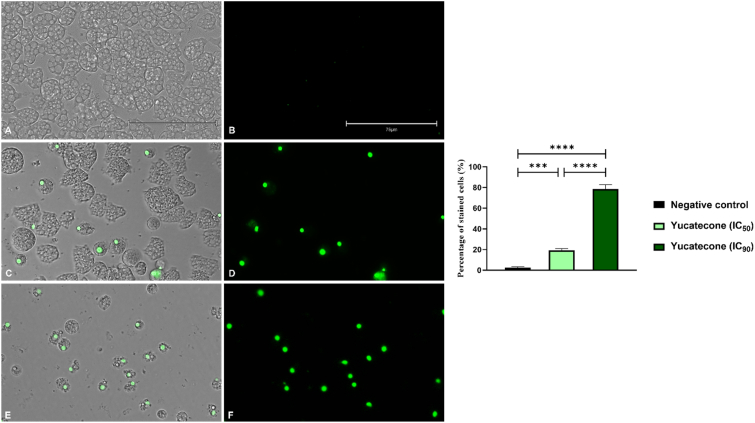
Permeation of the *N. fowleri* (ATCC 30808) trophozoites to the SYTOX® green vital dye after the treatment of the cells with the IC_50_ (**C** and **D**) and IC_90_ (**E** and **F**) of yucatecone. Treated cells show green fluorescence negative control (**A** and **B**). Images (x40) are representative of the cell population observed the EVOS M5000 Cell Imaging System, Life Technologies, Spain. (Scale bar: 75 μm). The bar graph shows the percentage of stained cells. It represents the mean value and the SD after performing the experiment in triplicate. Five different images (x40) in each experiment were analyzed and a one-way analysis of variance (ANOVA) was used to assess differences between the values ***p < 0.001; ****p < 0.0001 significance. The EVOS™ M5000 Software (Invitrogen by Thermo Fisher Scientific) was used to evaluate the stained cells. (For interpretation of the references to color in this figure legend, the reader is referred to the Web version of this article.)

The treatment of the amoebas with the yucatecone caused plasma membrane permeabilty damage as it can be observed in Fig. 3 and S18 where an intense green fluorescence inside the cells is shown. However, the cells kept the cytosolic content inside as it can be deduced from the absence of fluorescence in the media. Hence, the yucatecone induces the increasement of the membrane permeability without leading to the intracellular content release into the media, preventing the triggering of the immune response (Zhang et al., 2018). Control cells (incubated with culture media) show no fluorescence. The differences in the percentage of stained cells were statistically significant between the negative control and both groups of treated cells, as well as between the cells treated with the different concentrations of yucatecone (IC_50_ and IC_90_). On the other hand, SYTOX® green stain is considered a more sensitive and effective assay when speaking about membrane disruption and nucleic acid staining than the PI dye (Haase and Reed, 2002; Medwid et al., 2007; Roth et al., 1997). This could be the reason behind the positive result obtained in the SYTOX® green assay (Fig. 3) in contrast to the PI experiment result (Fig. 2).

### 3.5 Generation of intracellular ROS in treated cells

**Fig. 4:**
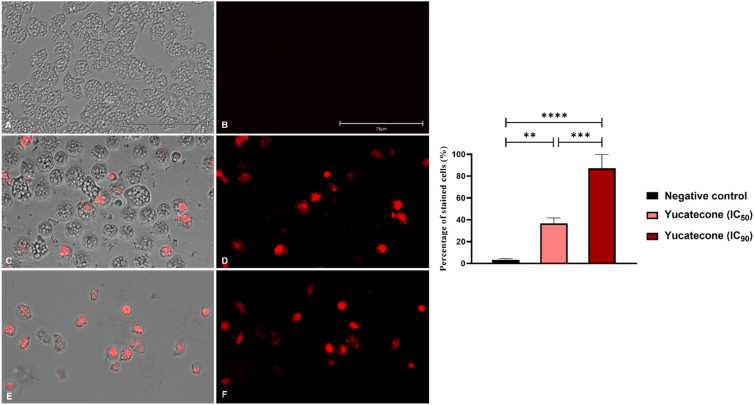
ROS detection after 24 h of incubation with the yucatecone at different concentrations; IC_50_ (**C** and **D**) and IC_90_ (**E** and **F**). An intense red fluorescence is shown after the treatment of the cells with evaluated molecule. Negative control (**A** and **B**). Images (x40) are representative of the cell population observed in the EVOS M5000 Cell Imaging System, Life Technologies, Spain. (Scale bar: 75 μm). The percentage of stained cells are represented in the graph, showing the mean value and the SD of three different assays. Percentage of stained cells were determined in the EVOS™ M5000 Software (Invitrogen by Thermo Fisher Scientific). A one-way analysis of variance (ANOVA) was used to assess differences between the values **p < 0.01; ***p < 0.001; ****p < 0.0001 significance. Five different images (x40) were processed each time. (For interpretation of the references to color in this figure legend, the reader is referred to the Web version of this article.)

The analysis of the ROS production with the CellROX Deep Red fluorescent assay indicated that cells incubated with yucatecone increased the intracellular ROS level production after 24 h. Fig. 4 and S19 show higher levels of red fluorescence in *N*. *fowleri* treated trophozoites in comparison with the negative control (non-treated cells). The treatment of the cells with the IC_50_ and the IC_90_ of the evaluated compound showed statistical differences in the percentage of stained cells comparing to the untreated amoebae.

### 3.6 Mitochondrial malfunction is presented by yucatecone treated amoebae

**Fig. 5:**
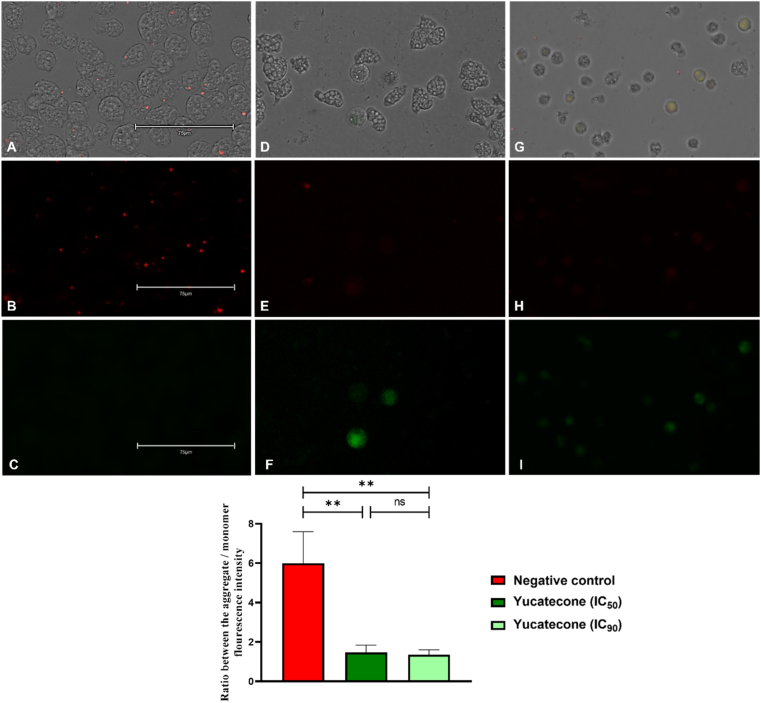
Mitochondrial membrane potential study of the *N. fowleri* (ATCC 30808) trophozoites after the treatment with the IC_50_ (**D**–**F**) and the IC_90_ of yucatecone (**G**–**I**). Negative control (**A**–**C**). In healthy cells, the JC-1 dye emmits red fluorescence since it accumulates as aggregates in the mitochondria (**B**, **E** and **H**). However, JC-1 remains in its monomeric form and emmits green fluorescence in yucatecone treated cells, due to the decrease in the mitochondrial membrane potential (**C**, **F** and **I**). Images are representative of the cell population observed in the EVOS M5000 Cell Imaging System, Life Technologies, Spain. (Scale bar: 75 μm). The graph represents the ratio between the fluorescence intensity of the aggregate and the monomeric form of the JC-1. Mean values and SD are represented after preforming the experiment in triplicate. The measurement of the fluorescence was carried out in the EVOS™ M5000 Software (Invitrogen by Thermo Fisher Scientific). Differences between the values were assessed using one-way analysis of variance (ANOVA) **p < 0.01, ns: non significance. (For interpretation of the references to color in this figure legend, the reader is referred to the Web version of this article.)

**Fig. 6:**
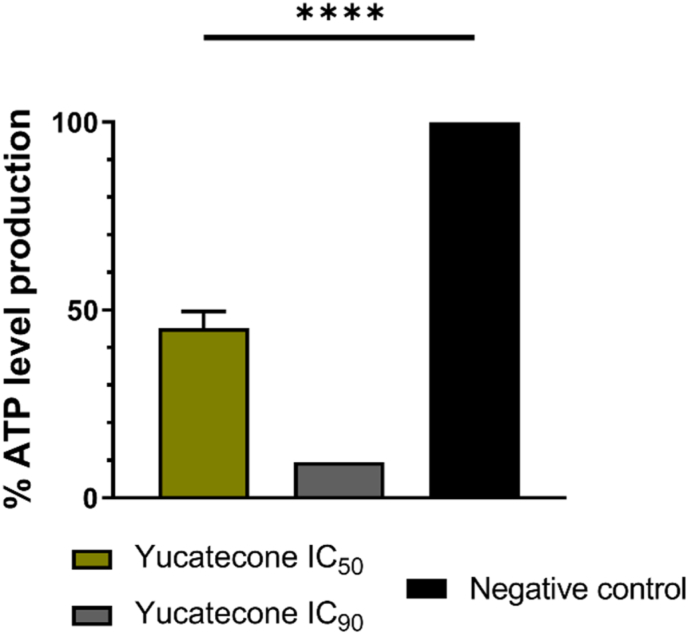
Percentage of ATP production of the trophozites incubated with the IC_50_ and the IC_90_ of the yucatecone compared to the untreated cells. Bars show the average results after three different assays. A decrease of a 54.80% and a 90.44%, respectively, in the ATP production was shown when the cells were treated. The standard deviation value of the results obtained in yucatecone IC_90_ treated cells was 0.0013%. Differences between the values were assessed using one-way analysis of variance (ANOVA). ****: p value < 0.0001.

The yucatecone induces the depolarization of the mitochondrial membrane potential of *N*. *fowleri* cells as it can be extracted from the Fig. 5 and S20 where the treated cells emit green fluorescence corresponding to the monomeric form of the JC-1 dye. Nevertheless, healthy cells (negative control, incubated in bactocasitone) emit red fluorescence as the JC-1 accumulates in the mitochondria and forms J-aggregates. Furthermore, the mitochondria disfunction was also checked by measuring the ATP level production. The evaluated oxasqualenoid decreased the ATP production a 54.80% when using the IC_50_ and a 90.44% with the IC_90_ comparing to the negative control (Fig. 6).

### 3.7 Structural analysis and *in silico* ADME/Tox properties of compounds **1**, **4** and **6**

Squalene-derived polyether metabolites are well known for their diverse biological properties, such as cytotoxicity, inhibition of Ser-Thr protein phosphatase 2A, VLA integrins activities and antifouling effects (Cen-Pacheco et al., 2011, 2015, 2018; Fernández et al., 2000). Additionally, these bioactive metabolites present a large diversity in ring size and functionalization as a consequence of a particular and interesting biosynthetic pathway (Fernández et al., 2000). As part of our research line dedicated to the search of bioactive molecules, five natural compounds, dehydrothyrsiferol (**1**), saiyacenols A and B (**2** and **3**) yucatecone (**4**), and 1,2-dehydropseudodehydrothyrsiferol (**5**) were reisolated from the red alga *Laurencia viridis*. Their structures were determined by the interpretation of NMR spectroscopic data and the relative configuration was established by NOESY correlations, *J*-based configurational analysis and chemical correlation (Cen-Pacheco et al., 2012, 2015, 2021). These compounds were evaluated for their antiamoebic activity, thus being the first report on their activity against *N*. *fowleri*.

**Fig. 7:**
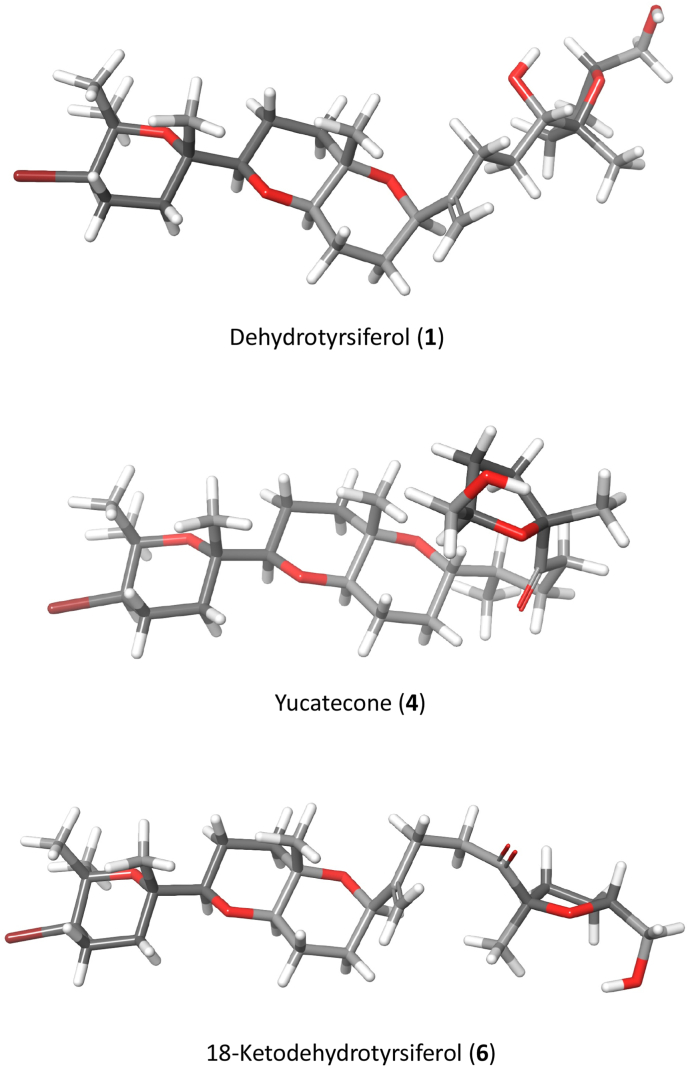
Minimized structures of the most stable conformer in solution of oxasqualenoids **1**, **4** and **6**. Data calculated with Schrödinger Release 2022–2: Maestro Version 13.0, Schrödinger, LLC, New York, NY, 2021(Schrödinger, 2021).

To establish a structural analysis, minimum energy conformers of compounds **1**, **4** and **6** were obtained using the conformational search panel from MacroModel, implemented on Schrödinger Suite 2021–4 (Chattaraj et al., 2006). For comparative purposes, the inactive compound **1** was included in this analysis. Systematic pseudo Monte Carlo method was used with OPLS4 force field and water solvent. The minimized structures of the most stable conformer of **1**, **4** and **6** in water solution are represented in Fig. 7.

The calculated values of energy (E) in aqueous solution of the most representative conformers of **1**, **4** and **6** are summarized in Table 2. Additionally, we developed an *in silico* ADME/Tox analysis. The prediction of the pharmacokinetic properties of compounds **1**, **4** and **6** were submitted by using the graphical interface Maestro and QikProp module of Schrödinger software (Schrödinger, 2021).

The molecular properties and solvation energies for each minima **1.1**, **4.1** and **6.1** were calculated with Jaguar module of Schrödinger Suites 2022–2 (Chattaraj et al., 2006; Schrödinger, 2021)(Chattaraj et al., 2006; Schrödinger, 2021) within the density functional theory (DFT) framework (Parr and Weitao, 1995a; Ziegler, 1991) using the B3LYP (Becke, 1988, 1993; Cen-Pacheco et al., 2011, 2015, 2018; Chattaraj et al., 2006; Fernández et al., 2000; Kohn et al., 1996; McIver and Komornicki, 1972; Becke, 1988, 1993; Kohn et al., 1996) along with the standard 6–31 G+ +* * basis set. All minima were fully characterized by harmonic frequency analysis (McIver and Komornicki, 1972). The extended results are included at the Supporting Information. Log *P* (*n*-octanol/water) is considered an illustrative descriptor to refer cell permeability (Soliman et al., 2021). The calculated value for dehydrotyrsiferol (**1**) (*log P* = 5.861) showed equivalent liposolubility values with yucatecone (**4**) (*log P* = 5.982) and 18-ketodehydrotyrsiferol (**6**) (*log P* = 5.786).

In this analysis, for all metabolites the estimated number of hydrogen bonds that would be donated and accepted by the solute to the water molecules in an aqueous solution 2.0 and 0.73 for dehydrotyrsiferol (**1**), respectively, whereas the active compounds yucatecone (**4**) and 18-ketodehydrotyrsiferol (**6**) showed values of 1.00 for donor and 0.76 for acceptor hydrogen bonds. The number of metabolic reactions of compounds **1** and **6** is 8, while in the case of yucatecone (**4**) is 6. All predicted metabolic reactions are analogous for the three compounds, except for **4** and **6**, that include α and β dehydrogenation at carbonyl position. Of note is difference of dipole moment (D) of compounds **4** and **6**, which showed values of 7.909 and 6.454, respectively, compared to 3.020 in the case of dehydrotyrsiferol (**1**). The predicted CNS activity, within a range -- to ++, is ± for **1**, and - in the case of oxasqualenoids **4** and **6**. All compounds showed a % Human Oral Absorption of above 92%. The number of violations of Lipinsky and Jorgensen's rules is 2 in all cases. Thus, it was observed that compounds dehydrotyrsiferol (**1**), yucatecone (**4**), and 18-ketodehydrotyrsiferol (**6**) were found to be within the limits of approved drug parameter range. Full Energy and ADME/Tox calculated data are available at the Supporting Information.

**Fig. 8:**
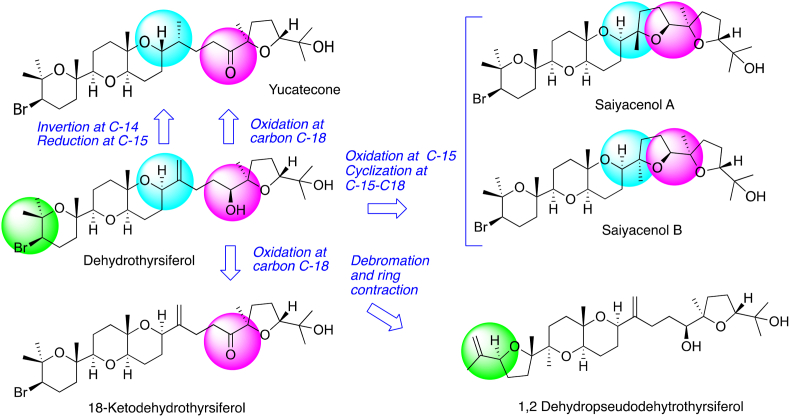
Structure–activity relationship of meroterpenoids from *Laurencia viridis* against *N fowleri* species.

Based on the above-mentioned results, in this study we have analyzed the SAR for *Laurencia* oxasqualenoids against *N*. *fowleri* (Fig. 8). Taking the inactive compound dehydrotyrsiferol (**1**) as starting target, we observe that the crucial structural motive to induce activity is the oxidation at C-18 from an alcohol to ketone group, observed in yucatecone (**4**) and 18-ketodehydrotyrsiferol (**6**). On the other hand, dehalogenation and ring contraction, inversion of C-14 stereocenter, and double bond reduction of C-15-C-28, as well as oxidation of C-15 followed by ring closure C-15-C-18 do not affect the activity.

## 4 Conclusion

We have analyzed the antiamoeboid effects of a family of natural oxasqualenoids **1**–**5** isolated from the red algae *Laurencia viridis*, and the semisynthetic analog 18-ketodehydrotyrsiferol (**6**). Despite the structural changes observed in this family of compounds, such as dehalogenation and ring contraction, inversion of C-14 stereocenter, and double bond reduction of C-15-C-28, oxidation at C-15 followed by ring closure C-15-C-18, it seems that the most significative feature to induce activity in *N*. *fowleri* is the presence of a ketone at C-18, as showed in the case of yucatecone (**4**) and 18-ketodehydrotyrsiferol (**6**), due to the punctual oxidation of compound 1 to 6 transform an inactive compound into a lead compound, with the lowest value of IC_50_ 12.70 μM. The assessment of *in silico* ADME/Tox analysis revealed that the active compounds showed good Human Oral Absorption and demonstrate that are found to be within the limit of approved drug parameter range.

In addition, yucatecone not only exhibited the highest selectivity index among the evaluated oxasqualenoids but it also showed to induce programmed cell death like process in treated amoebae which could presumably prevent the appearance of side effects. Therefore, yucatone can be considered as a promising molecule for the future PAM treatment, although further experiments need to be carried out.

## Author contributions

A.R.D.M., F.C.P., A.H.D. and J.J.F. processed the extract, conducted isolation of metabolites and spectroscopic analysis of the chemical compounds. J.L.M., A.R.D.M., F.C.P., J.E.P.B. conducted selection of *Laurencia* compounds. I.A.J., J.C.P, A.R.L., J.L.M. and I.S. performed the evaluation of amoebicidal activity and their interpretation assays of Programmed Cell Death. E.Q.M., A.R.D.M., and J.J.F. conducted molecular modeling, *in silico* ADME/Tox and SAR studies. Analysis and biological data compilation were performed by all authors. All authors contributed equally to the final version of the manuscript.

## Funding

This study was supported by the Consorcio Centro de Investigación Biomédica en Red (CIBER) de Enfermedades Infecciosas (CIBERINFEC), Instituto de Salud Carlos III, 28006 Madrid, Spain, Cabildo insular de Tenerife 2023-2028 and Ministerio de Sanidad, Spain, Proyecto Intramural Especial CSIC [Ref. 202280I032], and by the project No. 21/0587 funded by the Cabildo de Tenerife, Tenerife innova, Marco Estratégico de Desarrollo Insular (MEDI) and Fondo de Desarrollo de Canarias (FDCAN).

## Declaration of competing interest

The authors declare no conflict of interest.
