# Supplementary material for: Identification and characterization of novel marine oxasqualenoid yucatecone against Naegleria fowleri
Source: Int J Parasitol Drugs Drug Resist. 2023 May 29;22:61–71. doi: 10.1016/j.ijpddr.2023.05.004 (PMC10258243; doi:10.1016/j.ijpddr.2023.05.004)
Supplement: Multimedia component 1 [file mmc1.docx]

**Supporting Information**

**Identification and characterization of marine oxasqualenoid yucatecone against *Naegleria fowleri***

Iñigo Arberas-Jiménez^1,2^, Francisco Cen-Pacheco^3,4^, Javier Chao-Pellicer ^1,2,5^, Ines Sifaoui^1,2^, Aitor Rizo-Liendo^1,2^, Ezequiel Q. Morales^6^, Antonio H. Daranas^3,6^, Ana R. Díaz-Marrero,^3,6*^, José E. Piñero^1,2,5*^, José J. Fernández^3,7*^, Jacob Lorenzo-Morales^1,2,5^

^1^ Instituto Universitario de Enfermedades Tropicales y Salud Pública de Canarias (IUETSPC), Universidad de La Laguna (ULL), Avenida Astrofísico Francisco Sánchez s/n, 38203 La Laguna, Tenerife, Spain

^2^ Departamento de Obstetricia y Ginecología, Pediatría, Medicina Preventiva y Salud Pública, Toxicología, Medicina Legal y Forense y Parasitología, Universidad de La Laguna, Tenerife, Spain

^3^ Instituto Universitario de Bio-Orgánica Antonio González (IUBO AG), Universidad de La Laguna (ULL), Avenida Astrofísico Francisco Sánchez 2, 38206 La Laguna, Tenerife, Spain

^4^ Facultad de Bioanálisis, Universidad Veracruzana (UV), Agustín de Iturbide s/n, Centro, Veracruz, 91700, Mexico

^5^ Consorcio Centro de Investigación Biomédica (CIBER) de Enfermedades Infecciosas (CIBERINFEC), Instituto de Salud Carlos III, 28006 Madrid, Spain

^6^ Instituto de Productos Naturales y Agrobiología (IPNA), Consejo Superior de Investigaciones Científicas (CSIC), Avda. Astrofísico Francisco Sánchez 3, La Laguna, 38206, Tenerife, Spain

^7^ Departamento de Química Orgánica, Universidad de La Laguna (ULL), Avenida Astrofísico Francisco Sánchez s/n, 38203 La Laguna, Tenerife, Spain

* Correspondence: adiazmar@ipna.csic.es (A.R.D.-M.); jpinero@ull.edu.es (J.E.P); jjfercas@ull.edu.es (J.J.F.); jmlorenz@ull.edu.es (J.L.-M.)

**Table of contents**

| **Index** | |
| --- | --- |
| **Table S1**. NMR data of yucatecone (**4**) in CDCl_3_ at 298 K, 600 MHz. | **S4** |
| **Figure S1**. ^1^H-NMR spectrum of yucatecone (**4**) in CDCl_3_ at 298 K, 600 MHz. | **S5** |
| **Figure S2**. Section of the ^1^H-NMR decoupling spectrum of yucatecone (**4**) in CDCl_3_ at 298 K, 600 MHz. | **S6** |
| **Figure S3**. ^13^C-NMR spectrum of yucatecone (**4**) in CDCl_3_ at 298 K, 600 MHz. | **S7** |
| **Figure S4**. COSY spectrum of yucatecone (**4**) in CDCl_3_ at 298 K, 600 MHz. | **S8** |
| **Figure S5**. TOCSY spectrum of yucatecone (**4**) in CDCl_3_ at 298 K, 600 MHz. | **S9** |
| **Figure S6**. HSQC spectrum of yucatecone (**4**) in CDCl_3_ at 298 K, 600 MHz. | **S10** |
| **Figure S7**. HMBC spectrum of yucatecone (**4**) in CDCl_3_ at 298 K, 600 MHz. | **S11** |
| **Figure S8**. NOESY spectrum of yucatecone (**4**) in CDCl_3_ at 298 K, 600 MHz. | **S12** |
| **Figure S9**. HSQC-HECADE spectrum of yucatecone (**4**) in CDCl_3_ at 298 K, 600 MHz. | **S13** |
| **Figure S10**. *J*-HMBC spectrum of yucatecone (**4**) in CDCl_3_ at 298 K, 600 MHz. | **S14** |
| **Figure S11**. MS spectrum of yucatecone (**4**). | **S15** |
| **Table S2**. NMR data of 18-ketodehydrothyrsiferol (**6**) in CDCl_3_ at 298 K, 600 MHz. | **S16** |
| **Figure S12**. ^1^H-NMR spectrum of 18-ketodehydrothyrsiferol (**6**) in CDCl_3_ at 298 K, 600 MHz. | **S17** |
| **Figure S13**. ^13^C-NMR spectrum of 18-ketodehydrothyrsiferol (**6**) in CDCl_3_ at 298 K, 600 MHz. | **S18** |
| **Conformer Analysis** | **S19** |
| **Properties Predictions** | **S21** |
| **Figure S14.** Standard curve analysis for IC_50_ calculation of yucatecone against the trophozoite stage of *N. fowleri* ATCC®30808 ™ type strain | **S27** |
| **Figure S15.** Standard curve analysis for IC_50_ calculation of yucatecone against the trophozoite stage of *N. fowleri* ATCC®30808 ™ type strain | **S28** |
| **Figure S16.** Standard curve analysis for IC_50_ calculation of yucatecone against the cyst stage of *N. fowleri* | **S29** |
| **Figure S17.** Higher magnification of Figure 2 (x100). | **S30** |
| **Figure S18.** Higher magnification of Figure 3 (x100). | **S31** |
| **Figure S19.** Higher magnification of Figure 4 (x100). | **S32** |
| **Figure S20.** Higher magnification of Figure 5 (x100). | **S33** |

**Table S1**. NMR data of yucatecone (**2**) in CDCl_3_ at 298 K, 600 MHz.

| Position | δ_C_ | δ_H_ | Multiplicity | *^3^J*_H,H_ (Hz) |
| --- | --- | --- | --- | --- |
| 1 | 31.0 | 1.27 | s |  |
| 2 | 74.9 |  |  |  |
| 3 | 59.1 | 3.90 | dd | *4.0, 12.3* |
| 4 | 27.7 | 2.09 (α)  2.25 (β) | dddd  dddd | *3.5, 4.0, 4.3, 13.3*  *3.9, 12.0, 13.3, 13.5* |
| 5 | 37.2 | 1.55 (α)  1.80 (β) | ddd  ddd | *4.3, 13.5, 13.6*  *3.5, 3.9, 13.6* |
| 6 | 74.4 |  |  |  |
| 7 | 86.8 | 3.09 | dd | *2.4, 11.2* |
| 8 | 22.9 | 1.53 (α)  1.76 (β) | m  m |  |
| 9 | 37.7 | 1.50 (α)  1.70 (β) | m  m |  |
| 10 | 72.1 |  |  |  |
| 11 | 81.2 | 3.00 | dd | *3.8, 11.8* |
| 12 | 24.8 | 1.51 (β)  1.64 (α) | dddd  dddd | *3.2, 3.8, 4.0, 12.2*  *2.9, 11.8, 12.0, 12.2* |
| 13 | 28.2 | 1.37 (α)  1.65 (β) | dddd  dddd | *4.0, 11.7, 12.0, 12.0*  *2.1, 2.9, 3.2, 12.0* |
| 14 | 72.6 | 3.47 | ddd | *2.1; 4.6; 11.7* |
| 15 | 37.1 | 1.44 | m |  |
| 16 | 26.2 | 1.34  1.73 | m  m |  |
| 17 | 34.5 | 2.56  2.71 | ddd  ddd | *5.8, 9.7, 17.7*  *5.3, 9.9, 17.6* |
| 18 | 215.6 |  |  |  |
| 19 | 88.8 |  |  |  |
| 20 | 35.0 | 1.77  2.16 | m  m |  |
| 21 | 26.0 | 1.83 | m |  |
| 22 | 87.3 | 3.77 | dd | *6.2, 8.7* |
| 23 | 70.7 |  |  |  |
| 24 | 23.7 | 1.15 | s |  |
| 25 | 24.2 | 1.40 | s |  |
| 26 | 20.1 | 1.21 | s |  |
| 27 | 14.7 | 1.14 | s |  |
| 28 | 15.4 | 0.88 | d | *6.8* |
| 29 | 24.4 | 1.33 | s |  |
| 30 | 27.3 | 1.26 | s |  |

**Figure S1**. ^1^H-NMR spectrum of yucatecone (**4**) in CDCl_3_ at 298 K, 600 MHz.

**Figure S2**. Section of the ^1^H-NMR decoupling spectrum of yucatecone (**4**) in CDCl_3_ at 298 K, 600 MHz.


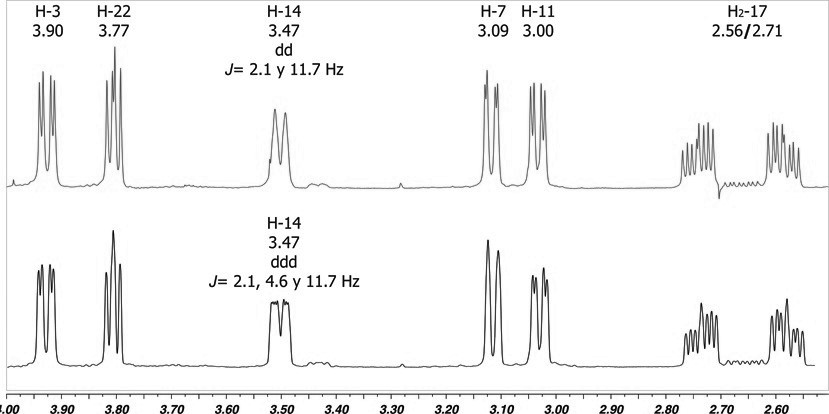


**Figure S3**. ^13^C-NMR spectrum of yucatecone (**4**) in CDCl_3_ at 298 K, 600 MHz.

**Figure S4**. COSY spectrum of yucatecone (**4**) in CDCl_3_ at 298 K, 600 MHz.


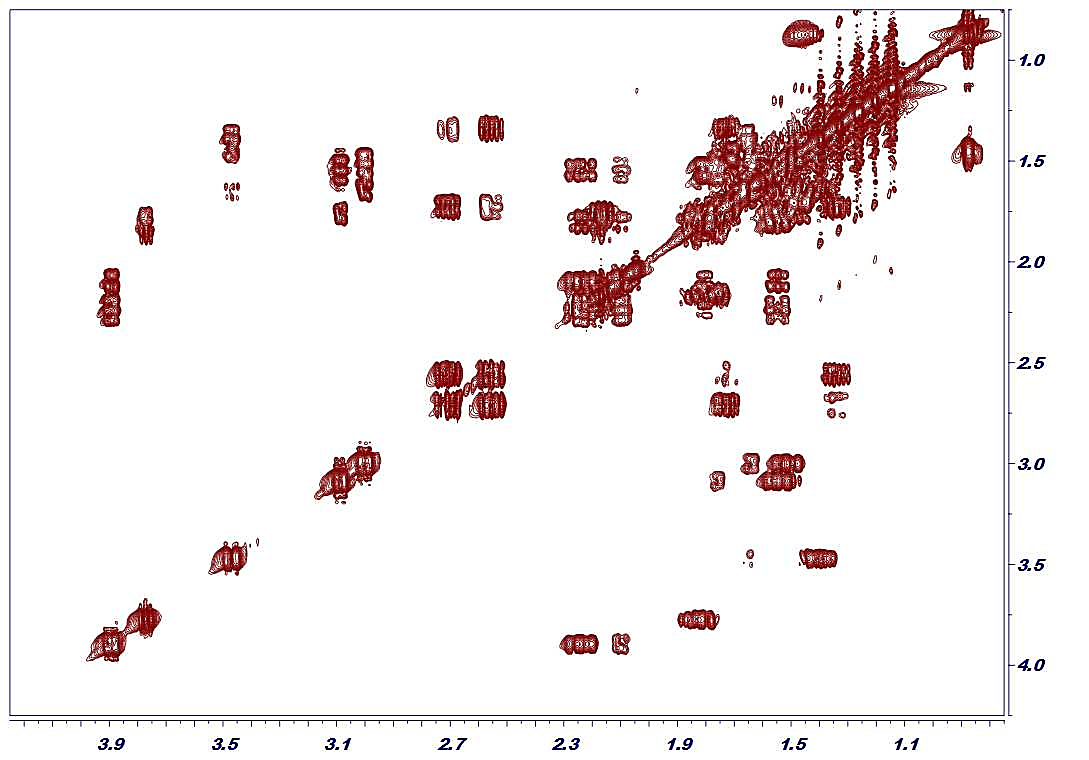


**Figure S5**. TOCSY spectrum of yucatecone (**4**) in CDCl_3_ at 298 K, 600 MHz.


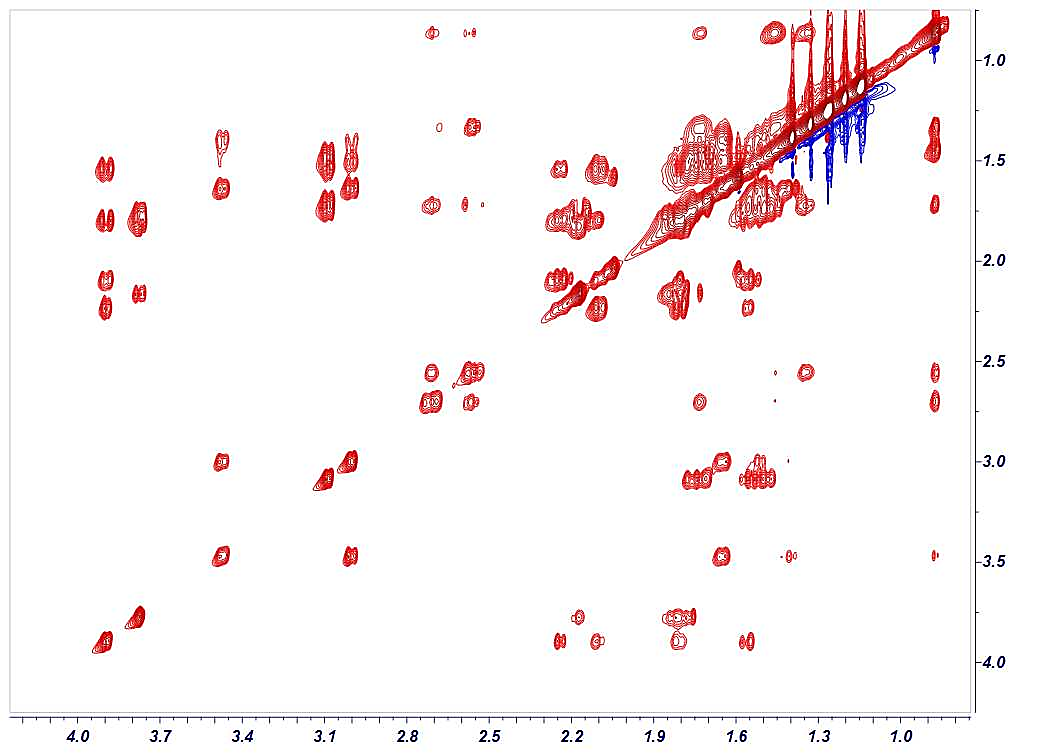


**Figure S6**. HSQC spectrum of yucatecone (**4**) in CDCl_3_ at 298 K, 600 MHz.


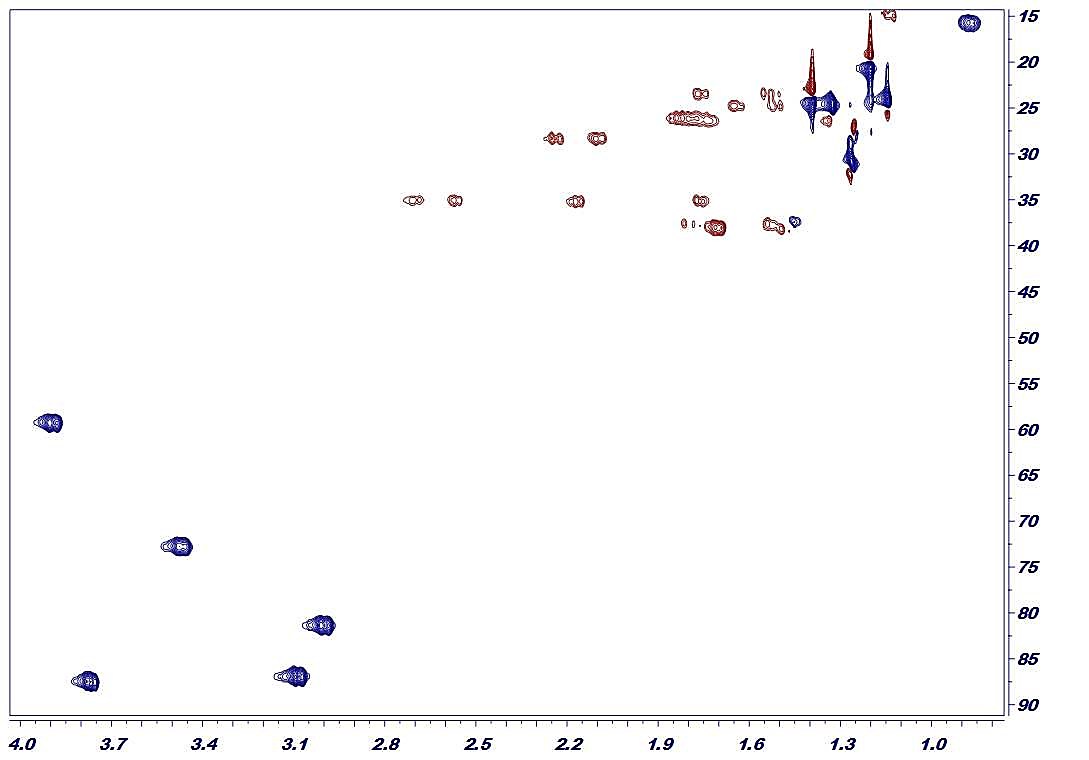


**Figure S7**. HMBC spectrum of yucatecone (**4**) in CDCl_3_ at 298 K, 600 MHz.


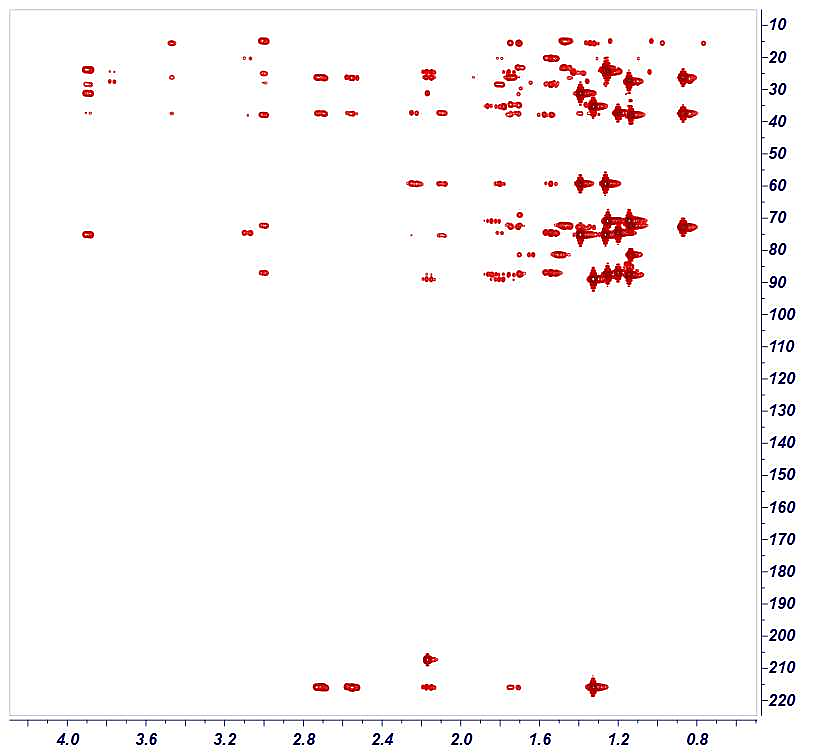


**Figure S8**. NOESY spectrum of yucatecone (**4**) in CDCl_3_ at 298 K, 600 MHz.


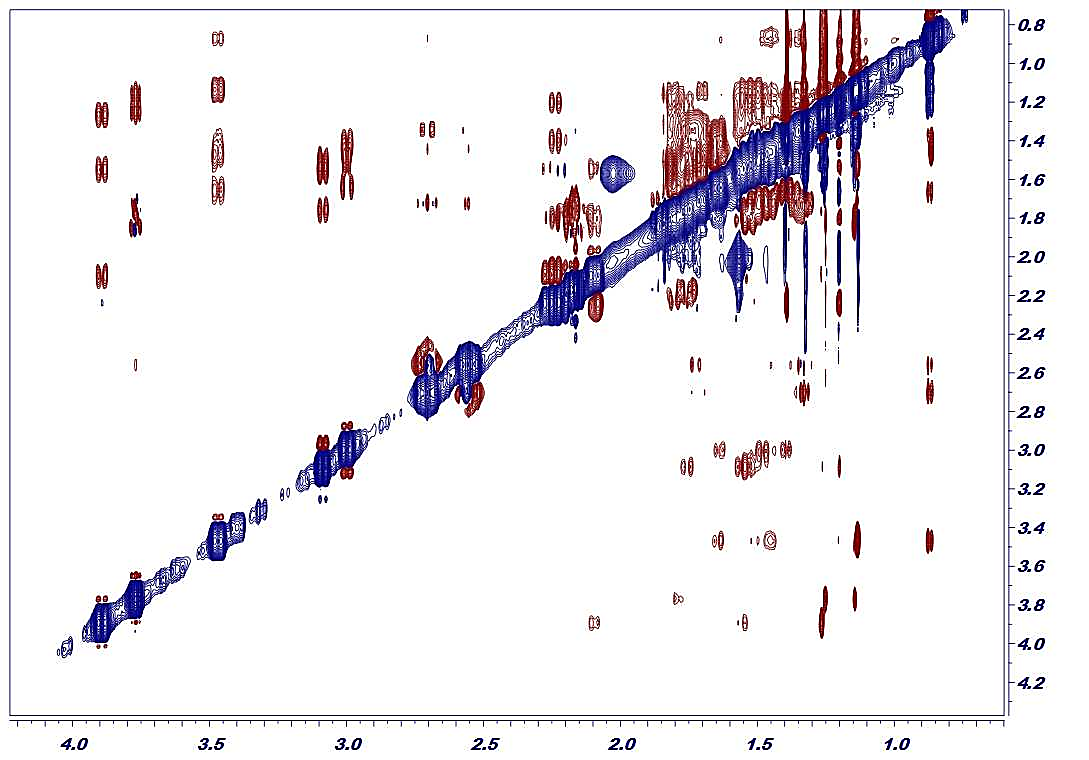


**Figure S9**. HSQC-HECADE spectrum of yucatecone (**4**) in CDCl_3_ at 298 K, 600 MHz.

**Figure S10**. *J*-HMBC spectrum of yucatecone (**4**) in CDCl_3_ at 298 K, 600 MHz.

**
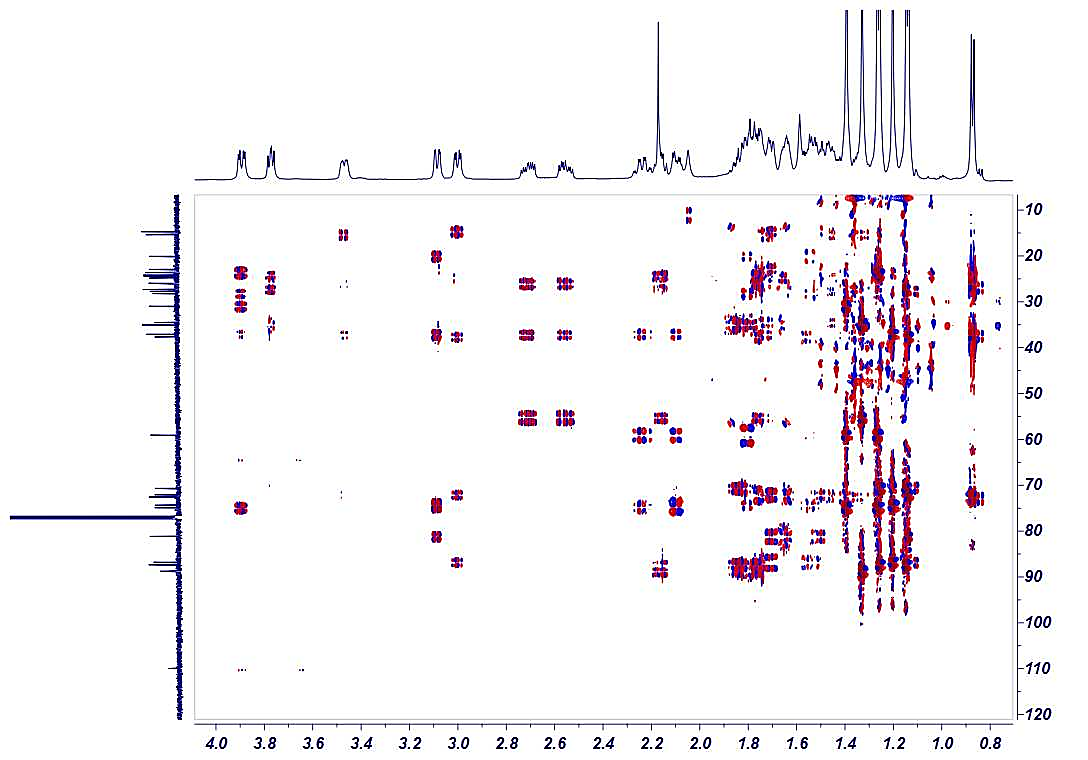
**

**Figure S11**. MS spectrum of yucatecone (**4**)


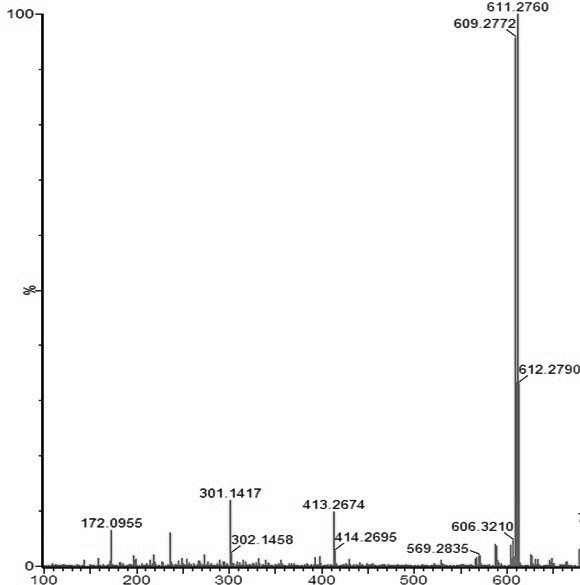


**Table S2**. NMR data of 18-ketodehydrothyrsiferol (**6**) in CDCl_3_ at 298 K, 600 MHz.

| Position | δ_C_ | δ_H_ | Multiplicity | *^3^J*_H,H_ (Hz) |
| --- | --- | --- | --- | --- |
| 1 | 31.1 | 1.27 | s |  |
| 2 | 75.0 |  |  |  |
| 3 | 59.1 | 3.89 | dd | *4.1, 12.6* |
| 4 | 28.0 | 2.13 (α)  2.26 (β) | m  m |  |
| 5 | 37.1 | 1.52 (α)  1.80 (β) | m  m |  |
| 6 | 74.3 |  |  |  |
| 7 | 86.7 | 3.08 | dd | *2.5, 11.0* |
| 8 | 23.0 | 1.49 (β)  1.76 (α) | m  m |  |
| 9 | 38.9 | 1.44 (α)  1.77 (β) | m  m |  |
| 10 | 72.7 |  |  |  |
| 11 | 78.8 | 3.42 | dd | *5.7, 11.3* |
| 12 | 21.9 | 1.60 (β)  1.81 (α) | m  m |  |
| 13 | 26.6 | 1.86 (β)  2.11 (α) | m  m |  |
| 14 | 72.5 | 4.29 | dd | *4.0, 8.1* |
| 15 | 151.4 |  |  |  |
| 16 | 29.8 | 2.12  2.40 | t |  |
| 17 | 35.5 | 2.70  2.93 | m  m |  |
| 18 | 215.3 |  | dd |  |
| 19 | 89.0 |  |  |  |
| 20 | 35.0 | 1.73  2.11 | m  m |  |
| 21 | 26.3 | 1.84 (2H) | m |  |
| 22 | 87.6 | 3.77 | dd | *6.0, 9.9* |
| 23 | 70.5 |  |  |  |
| 24 | 24.3 | 1.15 | s |  |
| 25 | 23.5 | 1.40 | s |  |
| 26 | 20.2 | 1.20 | s |  |
| 27 | 19.3 | 1.25 | s |  |
| 28 | 109.9 | 4.86  5.03 | bs  bs |  |
| 29 | 24.5 | 1.34 | s |  |
| 30 | 27.7 | 1.22 | s |  |

**Figure S12**. ^1^H-NMR spectrum of 18-ketodehydrothyrsiferol (**6**) in CDCl_3_ at 298 K, 600 MHz.

**
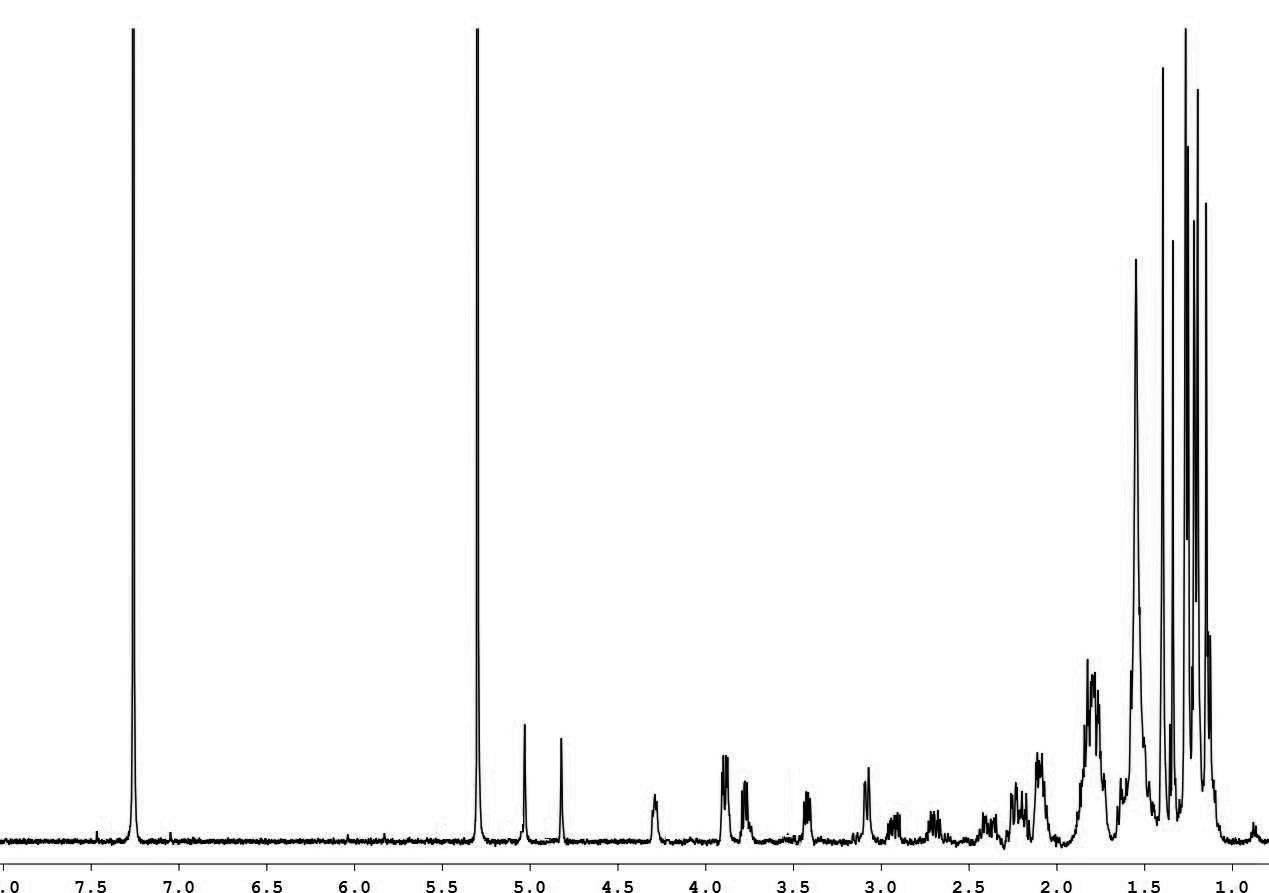
**

**Figure S13**. ^13^C-NMR spectrum of 18-ketodehydrothyrsiferol (**6**) in CDCl_3_ at 298 K, 600 MHz.

**
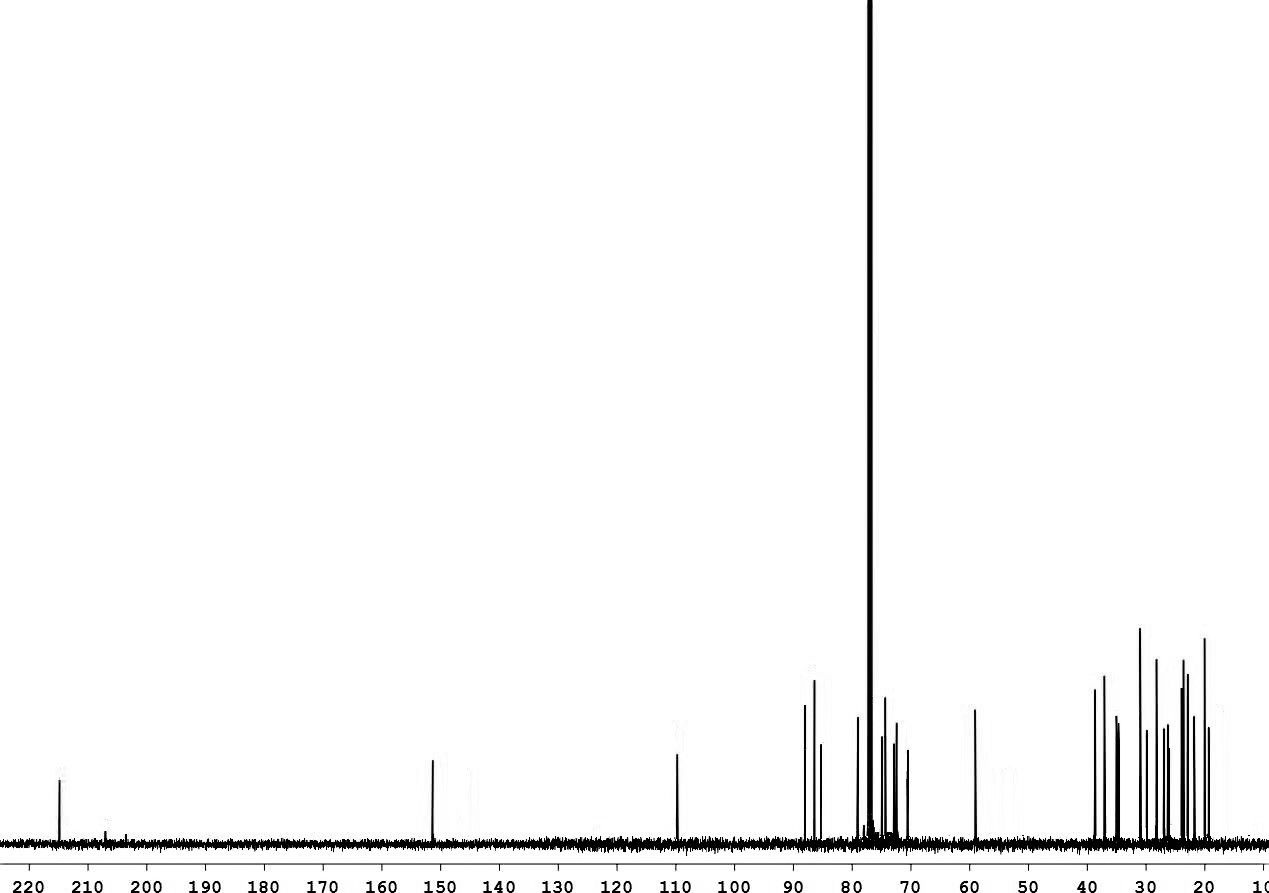
**

**Conformer Analysis**

From the conformational search described, 832 conformers of compound **1**, 2506 conformers of compound **4** and 2061 conformers of compound **6** were obtained and classified using X-Cluster software as implemented in Schrödinger Suites 2022-2 and reduced to the main conformers of each compounds: 4 conformers to **1**, 6 conformers to **6** and 10 conformers to **4.** That reduction in the number of conformers was made from the consideration of angles α, β and γ from **1** and **6**, and α, β, γ and δ from **4** on the side chain of the molecules.

**1** **4** **6**

From X-Cluster analysis we took a range on dihedral angles for each compound.

Dehydrotyrsiferol:

| 90 < α < 121 |  | 60 < β < 75 |  | 160 < γ < 180 |
| --- | --- | --- | --- | --- |
| 65 < α < 80 |  | ±160 < β < ±180 |  | 54 < γ < 75 |
| -6 > α < 30 |  | -96 < β < -60 |  | -90 < γ < -60 |
| -130 < α < -80 |  |  |  |  |

Yucatecone:

| ±140 < α < ±180 |  | 40 < β < 90 |  | ±175 < γ < ±180 |  | ±130 < δ < ±180 |
| --- | --- | --- | --- | --- | --- | --- |
| 50 < α < 65 |  | -120 < β < -45 |  | 60 < γ < 100 |  | 75 < δ < 100 |
| -70 < α < -50 |  | -180 < β < -140 |  | -90 < γ < -60 |  | -90 < δ < -65 |

18-Keto-dehydrotyrsiferol:

| 65 < α < 120 |  | 60 < β < 90 |  | ±130 < γ < ±180 |
| --- | --- | --- | --- | --- |
| -9 > α < 30 |  | -90 < β < -50 |  | 80 < γ < 102 |
| -140 < α < -60 |  | -180 < β < -150 |  | -80 < γ < -55 |

Conformers included in that dihedral range of values represent 84,45% of conformational population for compound **1**, 81,36% for compound **4** and 78,27% for compound **6**.

**Properties Predictions**

**Dehydrotyrsiferol (1)**

Primary Metabolites & Reactive FGs:

> Metabolism likely: alpha hydroxylation of cyclic ether

> Metabolism likely: alpha hydroxylation of cyclic ether

> Metabolism likely: allylic H -> alcohol

> Metabolism likely: alpha hydroxylation of cyclic ether

> Reactive FG: alkyl halide

> Metabolism likely: allylic H -> alcohol

> Metabolism likely: secondary alcohol -> ketone

> Metabolism likely: alpha hydroxylation of cyclic ether

> Metabolism likely: primary alcohol -> acid

Principal Descriptors: (Range 95% of Drugs)

Solute Molecular Weight = 559.580 ( 130.0 / 725.0)

Solute Dipole Moment (D) = 3.020 ( 1.0 / 12.5)

Solute Total SASA = 818.778 ( 300.0 /1000.0)

Solute Hydrophobic SASA = 685.675 ( 0.0 / 750.0)

Solute Hydrophilic SASA = 71.256 ( 7.0 / 330.0)

Solute Carbon Pi SASA = 10.016 ( 0.0 / 450.0)

Solute Weakly Polar SASA = 51.831 ( 0.0 / 175.0)

Solute Molecular Volume (A^3)= 1604.259 ( 500.0 /2000.0)

Solute vdW Polar SA (PSA) = 67.403 ( 7.0 / 200.0)

Solute No. of Rotatable Bonds= 8.000 ( 0.0 / 15.0)

Solute as Donor - Hydrogen Bonds = 2.000 ( 0.0 / 6.0)

Solute as Acceptor - Hydrogen Bonds = 7.350 ( 2.0 / 20.0)

Solute Globularity (Sphere = 1) = 0.809 ( 0.75 / 0.95)

Solute Ionization Potential (eV) = 9.871 ( 7.9 / 10.5)

Solute Electron Affinity (eV) = 0.325 ( -0.9 / 1.7)

Predictions for Properties:

QP Polarizability (Angstroms^3) = 53.007M ( 13.0 / 70.0)

QP log P for hexadecane/gas = 14.429M ( 4.0 / 18.0)

QP log P for octanol/gas = 24.257M ( 8.0 / 35.0)

QP log P for water/gas = 10.665M ( 4.0 / 45.0)

QP log P for octanol/water = 5.861 ( -2.0 / 6.5)

QP log S for aqueous solubility = -7.274 ( -6.5 / 0.5)*

QP log S - conformation independent = -7.748 ( -6.5 / 0.5)

QP log K hsa Serum Protein Binding = 1.150 ( -1.5 / 1.5)

QP log BB for brain/blood = -0.516 ( -3.0 / 1.2)

No. of Primary Metabolites = 8 ( 1.0 / 8.0)

Predicted CNS Activity (-- to ++) = +/-

HERG K+ Channel Blockage: log IC50 = -4.663 (concern below -5)

Apparent Caco-2 Permeability (nm/sec) = 2090 (<25 poor, >500 great)

Apparent MDCK Permeability (nm/sec) = 2110M (<25 poor, >500 great)

QP log Kp for skin permeability = -2.029 (Kp in cm/hr)

Jm, max transdermal transport rate = 0.000 (micrograms/cm^2-hr)

Lipinski Rule of 5 Violations = 2 (maximum is 4)

Jorgensen Rule of 3 Violations = 2 (maximum is 3)

% Human Oral Absorption in GI (+-20%) = 95 (<25% is poor)

Qual. Model for Human Oral Absorption = low (>80% is high)

A * indicates a violation of the 95% range. # stars = 1

An M indicates MW is outside training range.

5 of 1712 molecules most similar to **1**:

Name Similarity(%)

Hydrocortisone 82.98

Tropesin 82.83

Cilnidipine 77.04

Almitrine 76.51

Gliquidone 75.45

QP Breakdown (< for descriptor over training max)

log Po/w -log S

H-bond Donor -0.600 H-bond Donor -0.803

H-bond Acceptor -3.579 H-bond Acceptor -3.848

Volume 10.466 SASA 15.516

Ac x Dn^.5/SASA 0.563 Ac x Dn^.5/SASA 1.269

FISA -0.493 Rotor Bonds -1.302

Non-con amines 0.000 N Protonation 0.000

Non-con amides 0.000 Non-con amides 0.000

WPSA & PISA 0.209 WPSA 0.226

Constant -0.705 Constant -3.783

Total 5.861 Total 7.274

log BB log PMDCK

Hydrophilic SASA -0.725 Hydrophilic SASA -0.730

WPSA 0.127 WPSA 0.284

Rotor Bonds -0.482 0.000

N Protonation 0.000 Non-con amines 0.000

FOSA 0.000< COOH/SO3H acids 0.000

Constant 0.564 Constant 3.771

Total -0.516 Total 3.324

**Yucatecone (4)**

Primary Metabolites & Reactive FGs:

> Metabolism likely: alpha hydroxylation of cyclic ether

> Metabolism likely: alpha hydroxylation of cyclic ether

> Metabolism likely: alpha hydroxylation of cyclic ether

> Reactive FG: alkyl halide

> Metabolism likely: alpha, beta dehydrogenation at carbonyl

> Metabolism likely: alpha hydroxylation of cyclic ether

> Metabolism likely: primary alcohol -> acid

Principal Descriptors: (Range 95% of Drugs)

Solute Molecular Weight = 559.580 ( 130.0 / 725.0)

Solute Dipole Moment (D) = 7.909 ( 1.0 / 12.5)

Solute Total SASA = 822.868 ( 300.0 /1000.0)

Solute Hydrophobic SASA = 703.880 ( 0.0 / 750.0)

Solute Hydrophilic SASA = 67.221 ( 7.0 / 330.0)

Solute Carbon Pi SASA = 0.000 ( 0.0 / 450.0)

Solute Weakly Polar SASA = 51.768 ( 0.0 / 175.0)

Solute Molecular Volume (A^3)= 1619.783 ( 500.0 /2000.0)

Solute vdW Polar SA (PSA) = 71.474 ( 7.0 / 200.0)

Solute No. of Rotatable Bonds= 7.000 ( 0.0 / 15.0)

Solute as Donor - Hydrogen Bonds = 1.000 ( 0.0 / 6.0)

Solute as Acceptor - Hydrogen Bonds = 7.650 ( 2.0 / 20.0)

Solute Globularity (Sphere = 1) = 0.811 ( 0.75 / 0.95)

Solute Ionization Potential (eV) = 10.126 ( 7.9 / 10.5)

Solute Electron Affinity (eV) = 0.246 ( -0.9 / 1.7)

Predictions for Properties:

QP Polarizability (Angstroms^3) = 54.175M ( 13.0 / 70.0)

QP log P for hexadecane/gas = 14.230M ( 4.0 / 18.0)

QP log P for octanol/gas = 23.835M ( 8.0 / 35.0)

QP log P for water/gas = 9.631M ( 4.0 / 45.0)

QP log P for octanol/water = 5.982 ( -2.0 / 6.5)

QP log S for aqueous solubility = -7.419 ( -6.5 / 0.5)*

QP log S - conformation independent = -7.695 ( -6.5 / 0.5)

QP log K hsa Serum Protein Binding = 1.195 ( -1.5 / 1.5)

QP log BB for brain/blood = -0.413 ( -3.0 / 1.2)

No. of Primary Metabolites = 6 ( 1.0 / 8.0)

Predicted CNS Activity (-- to ++) = -

HERG K+ Channel Blockage: log IC50 = -4.597 (concern below -5)

Apparent Caco-2 Permeability (nm/sec) = 2282 (<25 poor, >500 great)

Apparent MDCK Permeability (nm/sec) = 2319M (<25 poor, >500 great)

QP log Kp for skin permeability = -2.086 (Kp in cm/hr)

Jm, max transdermal transport rate = 0.000 (micrograms/cm^2-hr)

Lipinski Rule of 5 Violations = 2 (maximum is 4)

Jorgensen Rule of 3 Violations = 1 (maximum is 3)

% Human Oral Absorption in GI (+-20%) = 96 (<25% is poor)

Qual. Model for Human Oral Absorption = low (>80% is high)

A * indicates a violation of the 95% range. # stars = 1

An M indicates MW is outside training range.

5 of 1712 molecules most similar to **4**:

Name Similarity(%)

Tropesin 85.47

Hydrocortisone 82.62

Gliquidone 79.09

Loperamide 78.69

Mibefradil 78.42

QP Breakdown (< for descriptor over training max)

log Po/w -log S

H-bond Donor -0.300 H-bond Donor -0.402

H-bond Acceptor -3.726 H-bond Acceptor -4.006

Volume 10.567 SASA 15.593

Ac x Dn^.5/SASA 0.412 Ac x Dn^.5/SASA 0.929

FISA -0.465 Rotor Bonds -1.140

Non-con amines 0.000 N Protonation 0.000

Non-con amides 0.000 Non-con amides 0.000

WPSA & PISA 0.197 WPSA 0.226

Constant -0.705 Constant -3.783

Total 5.982 Total 7.419

log BB log PMDCK

Hydrophilic SASA -0.682 Hydrophilic SASA -0.689

WPSA 0.127 WPSA 0.284

Rotor Bonds -0.422 0.000

N Protonation 0.000 Non-con amines 0.000

FOSA 0.000< COOH/SO3H acids 0.000

Constant 0.564 Constant 3.771

Total -0.413 Total 3.365

**18-ketodehydrotyrsiferol (6)**

Primary Metabolites & Reactive FGs:

> Metabolism likely: alpha hydroxylation of cyclic ether

> Metabolism likely: alpha hydroxylation of cyclic ether

> Metabolism likely: allylic H -> alcohol

> Metabolism likely: alpha hydroxylation of cyclic ether

> Reactive FG: alkyl halide

> Metabolism likely: allylic H -> alcohol

> Metabolism likely: alpha, beta dehydrogenation at carbonyl

> Metabolism likely: alpha hydroxylation of cyclic ether

> Metabolism likely: primary alcohol -> acid

Principal Descriptors: (Range 95% of Drugs)

Solute Molecular Weight = 557.564 ( 130.0 / 725.0)

Solute Dipole Moment (D) = 6.454 ( 1.0 / 12.5)

Solute Total SASA = 829.767 ( 300.0 /1000.0)

Solute Hydrophobic SASA = 671.844 ( 0.0 / 750.0)

Solute Hydrophilic SASA = 87.430 ( 7.0 / 330.0)

Solute Carbon Pi SASA = 18.717 ( 0.0 / 450.0)

Solute Weakly Polar SASA = 51.776 ( 0.0 / 175.0)

Solute Molecular Volume (A^3)= 1608.425 ( 500.0 /2000.0)

Solute vdW Polar SA (PSA) = 71.619 ( 7.0 / 200.0)

Solute No. of Rotatable Bonds= 7.000 ( 0.0 / 15.0)

Solute as Donor - Hydrogen Bonds = 1.000 ( 0.0 / 6.0)

Solute as Acceptor - Hydrogen Bonds = 7.650 ( 2.0 / 20.0)

Solute Globularity (Sphere = 1) = 0.800 ( 0.75 / 0.95)

Solute Ionization Potential (eV) = 9.791 ( 7.9 / 10.5)

Solute Electron Affinity (eV) = 0.278 ( -0.9 / 1.7)

Predictions for Properties:

QP Polarizability (Angstroms^3) = 53.899M ( 13.0 / 70.0)

QP log P for hexadecane/gas = 14.469M ( 4.0 / 18.0)

QP log P for octanol/gas = 23.636M ( 8.0 / 35.0)

QP log P for water/gas = 9.912M ( 4.0 / 45.0)

QP log P for octanol/water = 5.786 ( -2.0 / 6.5)

QP log S for aqueous solubility = -7.542 ( -6.5 / 0.5)*

QP log S - conformation independent = -7.653 ( -6.5 / 0.5)

QP log K hsa Serum Protein Binding = 1.159 ( -1.5 / 1.5)

QP log BB for brain/blood = -0.642 ( -3.0 / 1.2)

No. of Primary Metabolites = 8 ( 1.0 / 8.0)

Predicted CNS Activity (-- to ++) = -

HERG K+ Channel Blockage: log IC50 = -4.856 (concern below -5)

Apparent Caco-2 Permeability (nm/sec) = 1468 (<25 poor, >500 great)

Apparent MDCK Permeability (nm/sec) = 1439M (<25 poor, >500 great)

QP log Kp for skin permeability = -2.393 (Kp in cm/hr)

Jm, max transdermal transport rate = 0.000 (micrograms/cm^2-hr)

Lipinski Rule of 5 Violations = 2 (maximum is 4)

Jorgensen Rule of 3 Violations = 2 (maximum is 3)

% Human Oral Absorption in GI (+-20%) = 92 (<25% is poor)

Qual. Model for Human Oral Absorption = low (>80% is high)

A * indicates a violation of the 95% range. # stars = 1

An M indicates MW is outside training range.

5 of 1712 molecules most similar to **6**:

Name Similarity(%)

Tropesin 84.12

Gliquidone 79.74

Loperamide 79.35

Hydrocortisone 79.27

Brovanexine 78.04

QP Breakdown (< for descriptor over training max)

log Po/w -log S

H-bond Donor -0.300 H-bond Donor -0.402

H-bond Acceptor -3.726 H-bond Acceptor -4.006

Volume 10.493 SASA 15.724

Ac x Dn^.5/SASA 0.409 Ac x Dn^.5/SASA 0.922

FISA -0.605 Rotor Bonds -1.140

Non-con amines 0.000 N Protonation 0.000

Non-con amides 0.000 Non-con amides 0.000

WPSA & PISA 0.219 WPSA 0.226

Constant -0.705 Constant -3.783

Total 5.786 Total 7.542

log BB log PMDCK

Hydrophilic SASA -0.911 Hydrophilic SASA -0.896

WPSA 0.127 WPSA 0.284

Rotor Bonds -0.422 0.000

N Protonation 0.000 Non-con amines 0.000

FOSA 0.000< COOH/SO3H acids 0.000

Constant 0.564 Constant 3.771

Total -0.642 Total 3.158

**Figure S14**. Standard curve analysis for IC_50_ calculation using increasing doses of yucatecone against the trophozoite stage of *N. fowleri* type strain 30808™ determined by the alamarBlue® based colorimetric assay. Each data point represents the average value and the SD of three different assays.

**
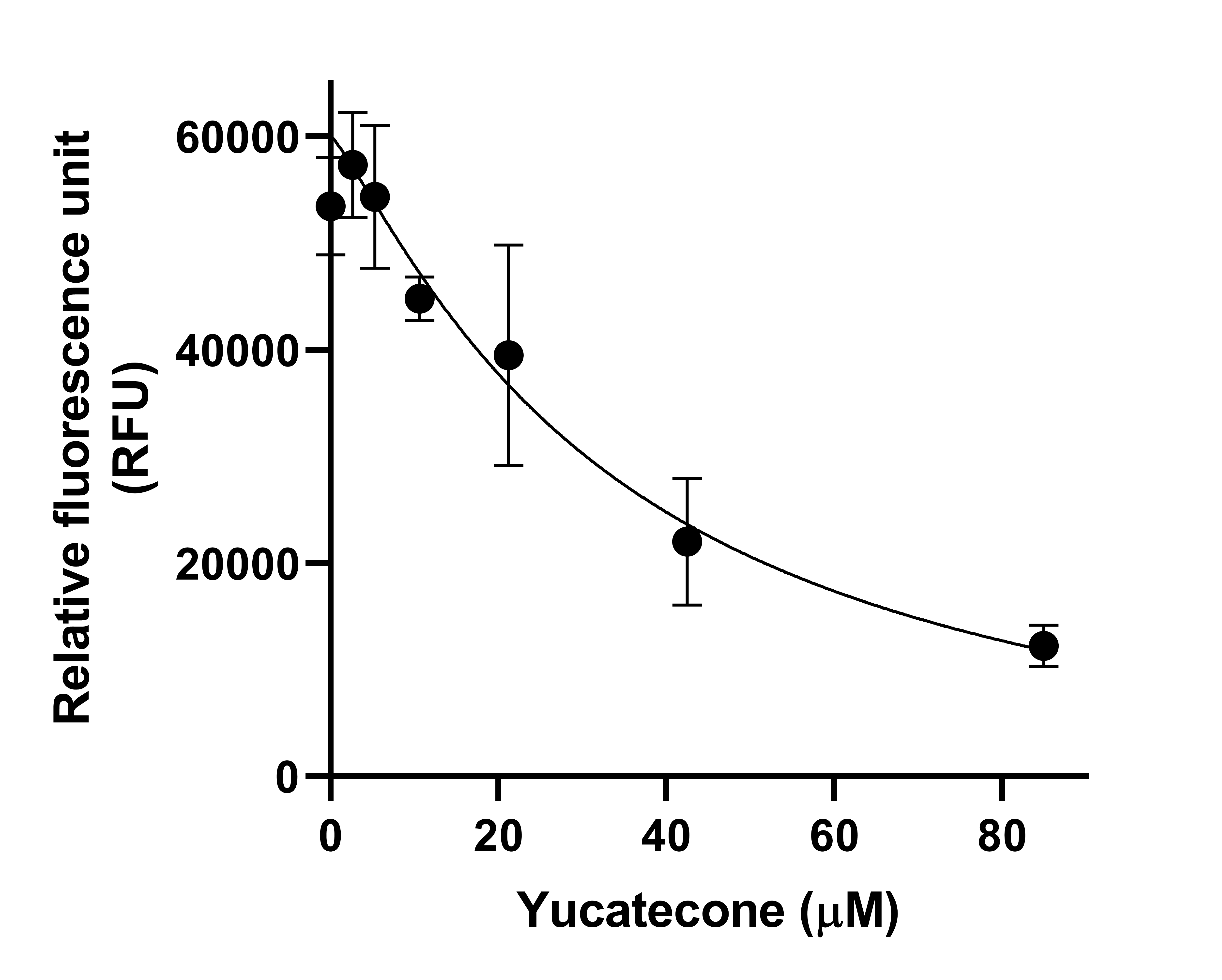
**

**Figure S15**. Standard curve analysis for IC_50_ calculation using increasing doses of yucatecone against the trophozoite stage of *N. fowleri* ATCC®30808™ type strain determined by the alamarBlue® based colorimetric assay. Each data point represents the average value and the SD of three different assays.

**
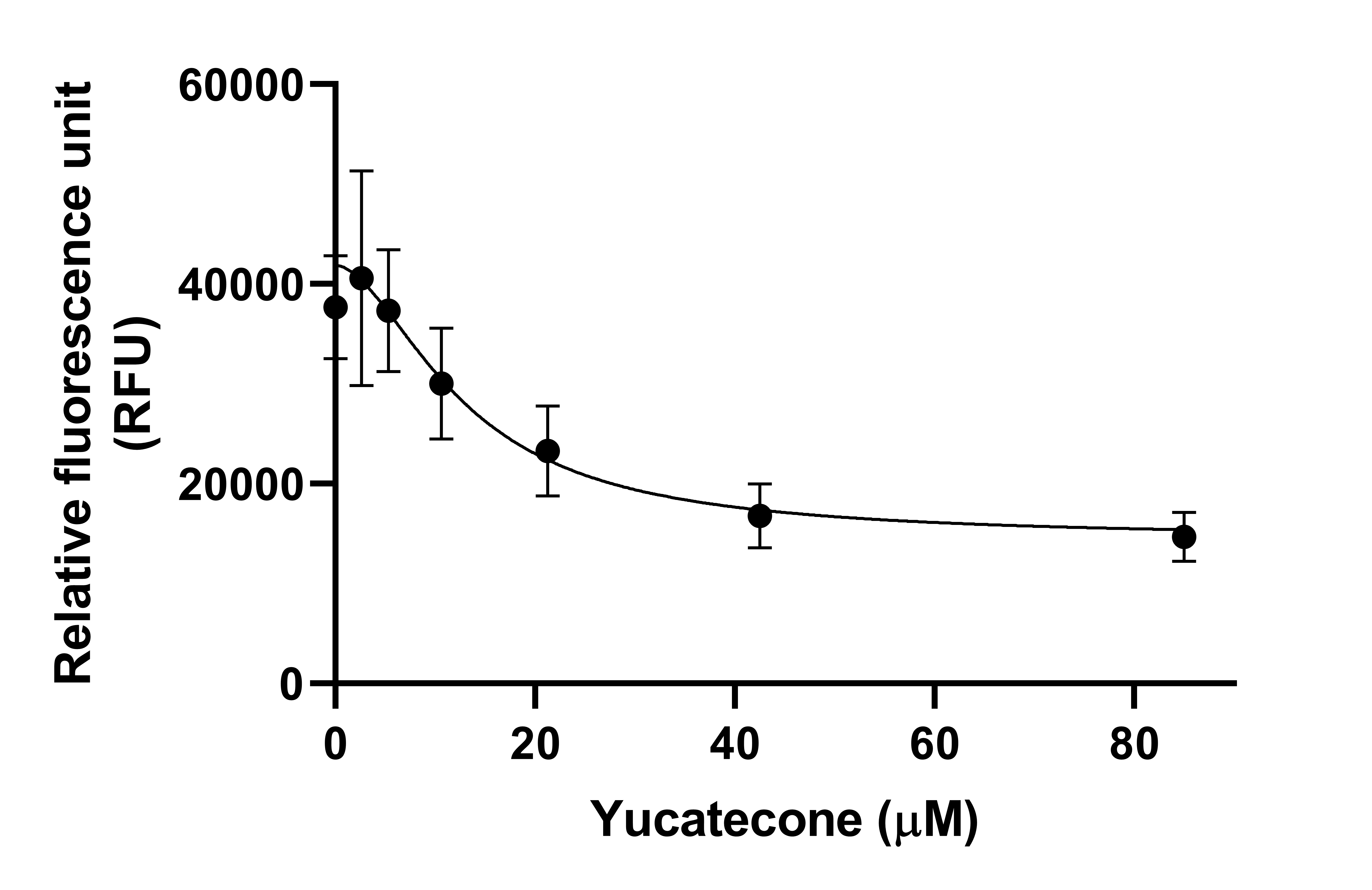
**

**Figure S16**. Standard curve analysis for IC_50_ calculation using increasing doses of yucatecone against the cyst stage of *N. fowleri* ATCC®30215™ type strain determined by the alamarBlue® based colorimetric assay. Each data point represents the average value and the SD of three different assays.

**
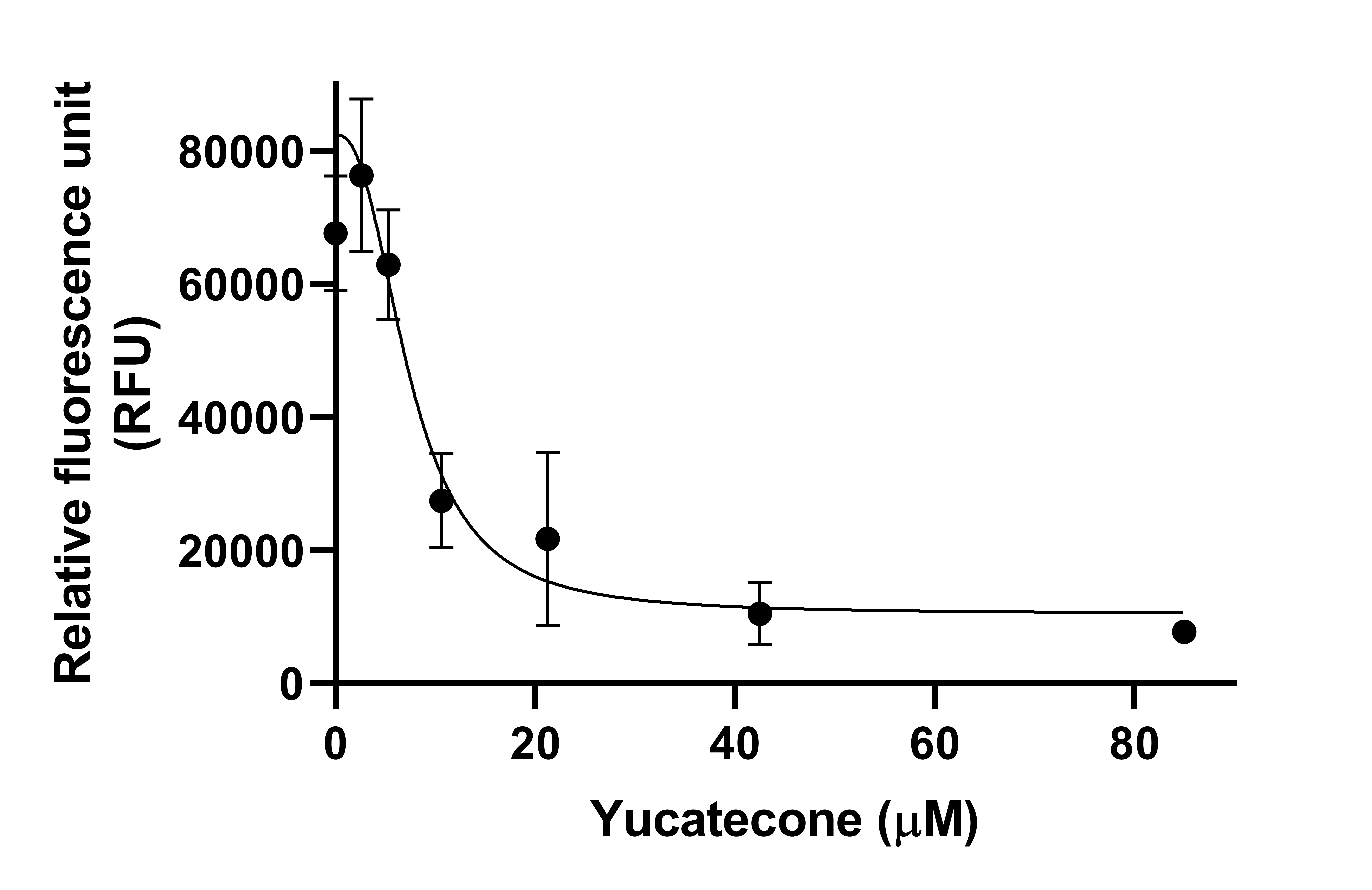
**

**Figure S17**. Higher magnification of Figure 2 (x100). (D-F) images show early apoptotic cells with the condensed chromatin shown as blue fluorescence (E). Overlay channel (A and D); Hoechst channel (B and E), and propidium iodide channel (C and F). Images are representative of the cell population observed in the performed experiments. Images were obtained using an EVOS M500 Cell Imaging System, Life Technologies, Spain. (Scale bar: 20 µm).

**
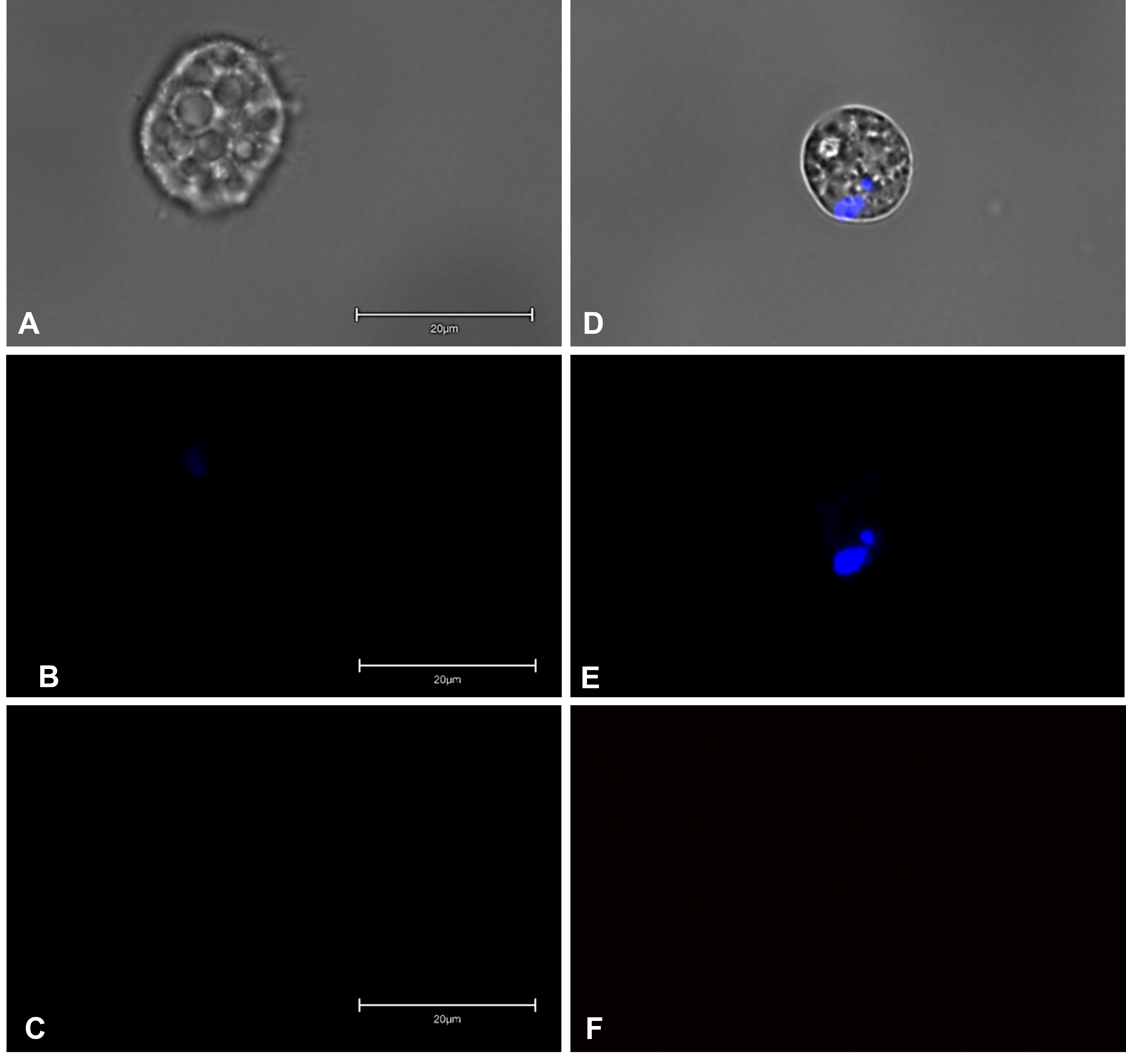
**

**Figure S18.** Higher magnification of Figure 3 (x100). Treated cells show intense green fluorescence (**C** and **D**), negative control (**A** and **B**). Overlay channel (**A** and **C**) and Sytox green channel (**B** and **D**). Images are representative of the cell population observed in the performed experiments. Images were obtained using an EVOS M500 Cell Imaging System, Life Technologies, Spain. (Scale bar: 20 µm).

**
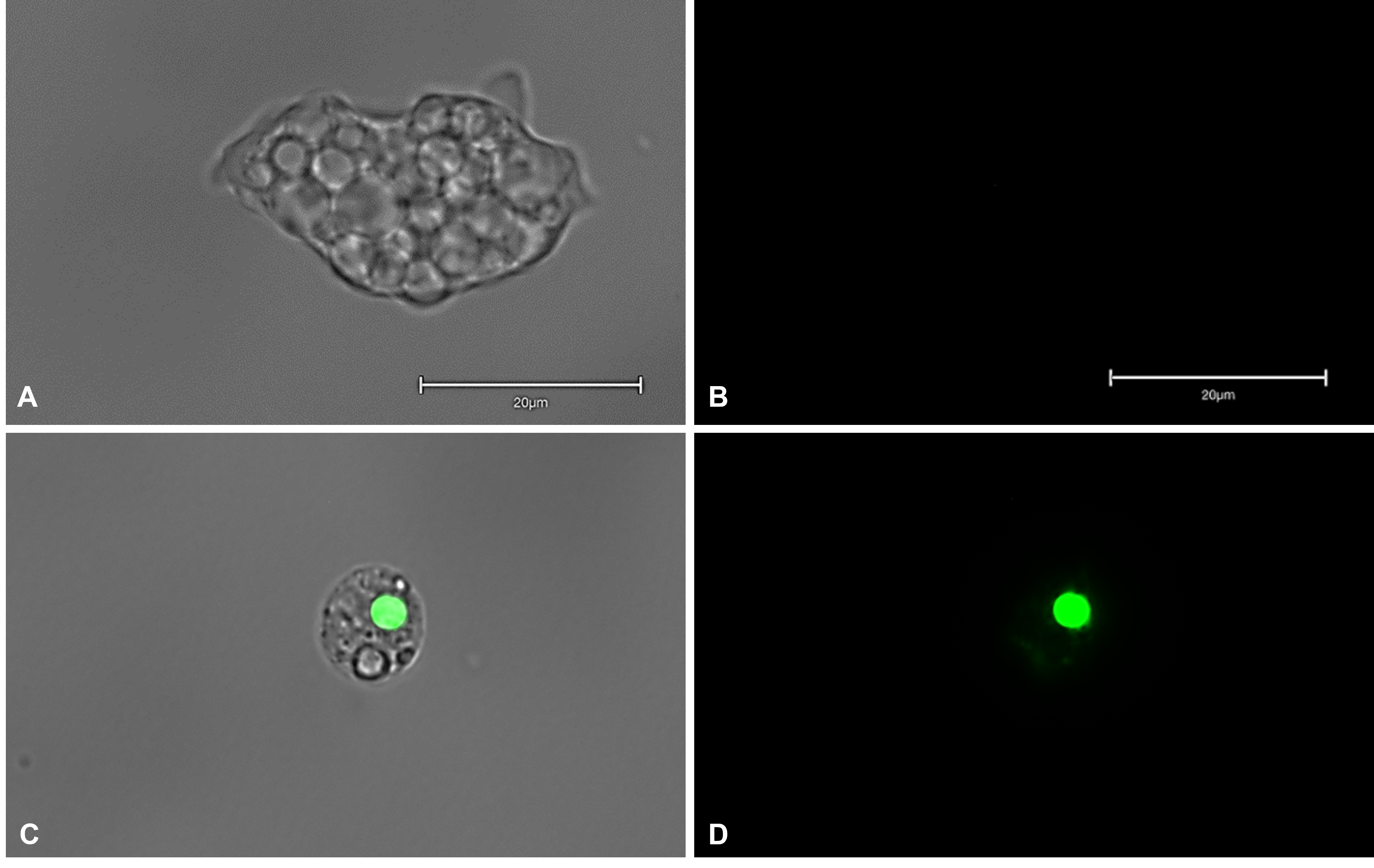
**

**Figure S19.** Higher magnification of Figure 4 (x100). Cells treated with the IC_90_ of yucatecone showing red fluorescence that corresponds to the presence of ROS (**C** and **D**), negative control (**A** and **B**). Overlay channel (**A** and **C**) and CellROX® channel (**B** and **D**). Images are representative of the cell population observed in the performed experiments. Images were obtained using an EVOS M500 Cell Imaging System, Life Technologies, Spain. (Scale bar: 20 µm).

**
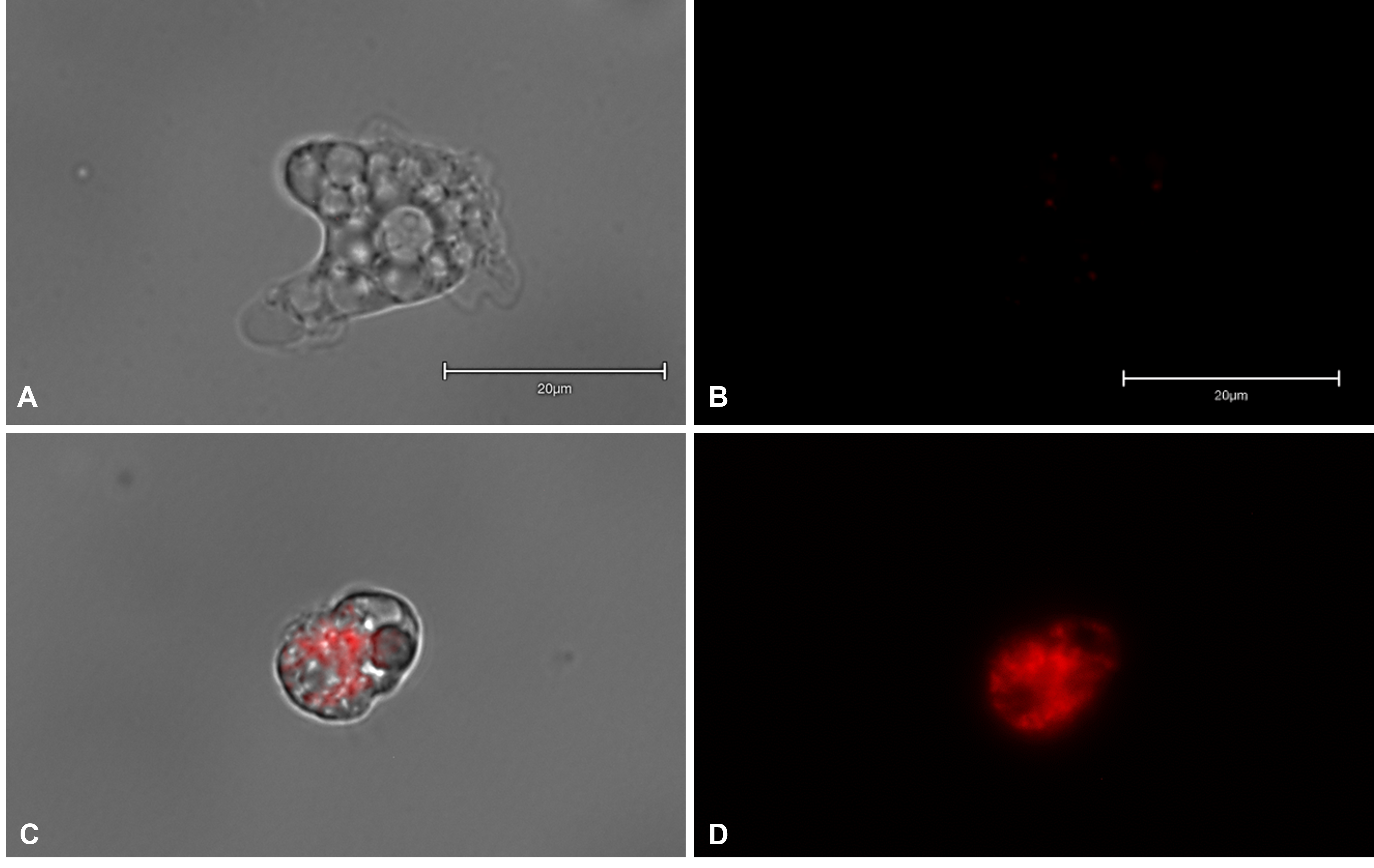
**

**Figure S20**. Higher magnification of Figure 5 (X100). Overlay channel (A and D). Red channel (B and E) where JC-1 is shown in the aggregated form. Green channel (C and F) showing JC-1 dye in its monomeric form. Images are representative of the cell population observed in the performed experiments. Images were obtained using an EVOS M5000 Cell Imaging System, Life Technologies, Spain. (Scale bar: 20 µm)

**
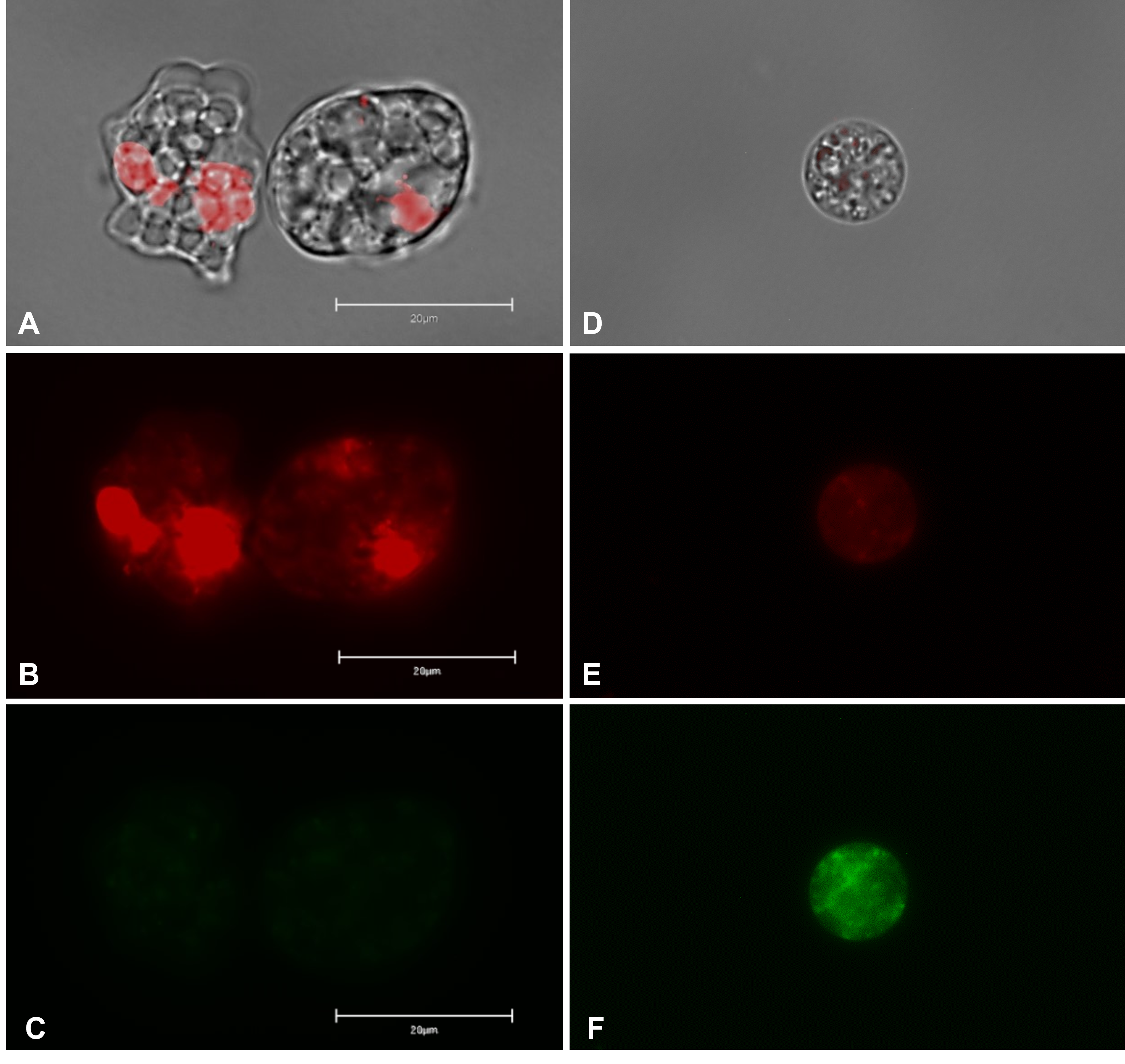
**
